# The effect of burst suppression on cerebral blood flow and autoregulation: a scoping review of the human and animal literature

**DOI:** 10.3389/fphys.2023.1204874

**Published:** 2023-06-07

**Authors:** A. Zohaib Siddiqi, Logan Froese, Alwyn Gomez, Amanjyot Singh Sainbhi, Kevin Stein, Kangyun Park, Nuray Vakitbilir, Frederick A. Zeiler

**Affiliations:** ^1^ Department of Medicine, Rady Faculty of Health Sciences, University of Manitoba, Winnipeg, MB, Canada; ^2^ Biomedical Engineering, Faculty of Engineering, University of Manitoba, Winnipeg, MB, Canada; ^3^ Department of Human Anatomy and Cell Science, Rady Faculty of Health Sciences, University of Manitoba, Winnipeg, MB, Canada; ^4^ Undergraduate Medicine, Rady Faculty of Health Sciences, University of Manitoba, Winnipeg, MB, Canada; ^5^ Section of Neurosurgery, Department of Surgery, Rady Faculty of Health Sciences, University of Manitoba, Winnipeg, MB, Canada; ^6^ Department of Clinical Neuroscience, Karolinska Institute, Stockholm, Sweden; ^7^ Division of Anaesthesia, Department of Medicine, Addenbrooke’s Hospital, University of Cambridge, Cambridge, United Kingdom

**Keywords:** cerebral blood flow (CBF), cerebrovascular physiology, cerebral autoregulation (CA), neuroanaesthesia, systematic review, burst suppression

## Abstract

**Background:** Burst suppression (BS) is an electroencephalography (EEG) pattern in which there are isoelectric periods interspersed with bursts of cortical activity. Targeting BS through anaesthetic administration is used as a tool in the neuro-intensive care unit but its relationship with cerebral blood flow (CBF) and cerebral autoregulation (CA) is unclear. We performed a systematic scoping review investigating the effect of BS on CBF and CA in animals and humans.

**Methods:** We searched MEDLINE, BIOSIS, EMBASE, SCOPUS and Cochrane library from inception to August 2022. The data that were collected included study population, methods to induce and measure BS, and the effect on CBF and CA.

**Results:** Overall, there were 66 studies that were included in the final results, 41 of which examined animals, 24 of which examined humans, and 1 of which examined both. In almost all the studies, BS was induced using an anaesthetic. In most of the animal and human studies, BS was associated with a decrease in CBF and cerebral metabolism, even if the mean arterial pressure remained constant. The effect on CA during periods of stress (hypercapnia, hypothermia, etc.) was variable.

**Discussion:** BS is associated with a reduction in cerebral metabolic demand and CBF, which may explain its usefulness in patients with brain injury. More evidence is needed to elucidate the connection between BS and CA.

## 1 Introduction

**FIGURE 1:**
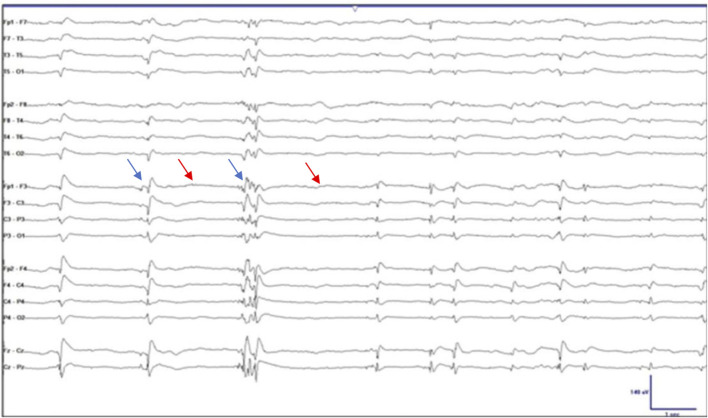
Electroencephalographic burst suppression. The blue arrows indicate the spikes or “bursts” and the red arrows indicate the epochs of “suppression.” Taken and adapted with permission from Ng et al.,2015.

“Burst suppression” (BS) is an electroencephalography (EEG) phenomenon in which there is continuous alternation between epochs of suppressed activity, evidenced by “flattening” or slowing of the wave form, and high-voltage sharp waves, or “spikes” on the wave form (Amzica, 2009; Amzica, 2015; Shanker et al., 2021). One method of identifying BS is purely by visualization (see Figure 1). According to the American Clinical Neurophysiological Society, when an EEG shows 50%–99% suppression of the waveform, it can be termed BS (Ma and Bebawy, 2022). Another, more accurate method is through identification of the burst-suppression ratio (BSR). The BSR can be obtained by dividing the total duration of suppression by the total epoch time and then multiplying the result by 100; in other words, if a waveform was completely suppressed or “isoelectric,” the BSR would be 100% and if there was absolutely no suppression the BSR would be 0%. Yet another method is through power spectral analysis (PSA) in which frequency-domain signal analytic approaches are used to convert EEG signals in which amplitude is plotted against frequency. This method quantifies the amplitude of each frequency component and is a more objective measure of the EEG and BS (Liu et al., 2021). By performing PSA, spectrograms can be generated which can provide visually striking displays of deep sedation (Shanker et al., 2021).

BS has been seen as a harbinger of severe neurological injury as in coma, cerebral anoxia, or infantile encephalopathy (Amzica, 2015; Shanker et al., 2021; Ma and Bebawy, 2022). In one retrospective review of 101 patients in a coma, BS with identical bursts/spikes was associated with poor outcome with a specificity and positive predictive value of 100% (Hofmeijer et al., 2014). However, interestingly, BS has also been used as a method of neuroprotection, usually using anaesthetic agents. It is hypothesized that BS leads to a state of cortical inactivation, decreasing cerebral metabolism and oxygen demand (CMR_O2_) and thereby can be protective during times of cerebral stress such as during cardiac surgery, cerebral aneurysm surgery, and carotid surgery (Ma and Bebawy, 2022).

BS may also be protective for patients with traumatic brain injury (TBI) (Frohlich et al., 2021). As cerebral perfusion pressure (CPP) is equal to the difference between the mean arterial pressure (MAP) and intracranial pressure (ICP), it is possible that BS can be neuroprotective through decreasing ICP in patients with brain injury (Mount and Das, 2022). In a systematic review, Zeiler et al., demonstrated Oxford level 2b and GRADE C evidence to support that BS reduces ICP but does not have any effect on ICP in severe TBI (Zeiler et al., 2017). Given that ICP reduction happened no matter which anaesthetic was used, the authors hypothesized that it was BS that caused the ICP reduction and not the anaesthetic itself. One possibility is that BS leads to the reduction of ICP by reducing cerebral blood flow (CBF). The normal CBF ranges from 45 to 50 mL/100 g/min, being higher in grey matter and lower in white matter (Tameem and Krovvidi, 2013). When cerebral blood vessels dilate (due to hypercapnia, drugs, or a variety of other triggers) the resulting CBF increases, increasing the amount of cerebral blood volume, and concurrently, the ICP.

CBF is tightly controlled through a variety of mechanisms (Tameem and Krovvidi, 2013). The term “cerebral autoregulation” (CA) applies to the capacity of the cerebral blood vessels to alter their resistance to maintain constant CBF in the face of a constantly changing MAP. When there are changes in MAP, there are changes in the smooth muscle of the cerebral vasculature through nitric oxide and endothelium-derived relaxing factors (Tameem and Krovvidi, 2013). Classically, autoregulation occurs between a MAP of 50 and 150 mmHg and outside of this range, CBF becomes significantly dependent on MAP and changes accordingly; this is known as the “Lassen Curve” (11). Different disease states, from hypertension to hydrocephalus can shift this curve, thereby increasing the risk of pressure-passive neural injury, even at a higher MAP (12). CBF is also highly coupled to neuronal activity, a process known as “flow-metabolism coupling”. An increase in neuronal activity leads to an increase in CMR_O2_ and cerebral metabolic rate for glucose (CMR_glu_) and there are proportional increases in CBF (Tameem and Krovvidi, 2013). CMR_O2_ can be calculated by multiplying the CBF and the difference in oxygen concentration in the cerebral veins and arteries (arteriovenous difference in oxygen). To measure autoregulation, it is essential to measure changes in CBF while keeping all other factors such as partial pressure of CO_2_ and cerebral metabolic rate (CMR) constant.

Many anaesthetics decrease CBF, but it remains unclear if it is the anaesthetic itself or the BS that causes a reduction in CBF; that is to say, is the depression of CMR_O2_ responsible for the decrease in CBF through flow-metabolism coupling or the direct effect of the anaesthetic on the cerebral vasculature? Further, it is unclear whether anaesthetics effect autoregulation in addition to the CBF. We sought to conduct a systematic scoping review on the effect that BS, induced primarily by anaesthesia, has on CBF and autoregulation in animals and humans.

## 2 Materials and methods

Using the methodology outlined in the Cochrane Handbook for Systematic Reviews, we performed a systematically conducted scoping review of the available literature (Cochrane Handbook, 2023). Reporting was done in accordance with the Preferred Reporting Items for Systematic Reviews and Meta-Analysis (PRISMA) (Tricco et al., 2018). The methodology and search strategy followed are similar to previous systematic scoping reviews conducted by our group (Froese et al., 2020a; Sainbhi et al., 2021; Batson et al., 2022; Gomez et al., 2022). Review objectives and search strategy were conceptualized by the primary (AS, LF) and senior (FZ.) authors.

### 2.1 Search question, population, inclusion, and exclusion criteria

The question evaluated for this systematic review was as follows: What is the effect of BS on CBF and CA in animals and humans?

BS was defined as an EEG pattern characterized by alternating periods of isoelectricity and active oscillations or “spikes” (2). Note, for the present study, “isoelectricity” was considered synonymous with BS as they both indicate a state of profound cerebral inactivity. BS could be induced by anaesthesia or by brain injury.

The measures of CBF that were included in the search were the following: measuring blood flow in the superior sagittal sinus, Xenon133 clearance, MS techniques (radiolabelled, florescent, or coloured), laser Doppler flowmetry, thermal diffusion flowmetry, positron emission tomography (PET) imaging, perfusion weighted magnetic resonance imaging (PW-MRI), arterial spin labeling magnetic resonance imaging (ASL-MRI), functional magnetic resonance imaging (fMRI) and blood-oxygen-level-dependent (BOLD), perfusion computed tomography (CTP), xenon-enhanced perfusion computed tomography (Xe-CT), single-photon emission computed tomography (SPECT), near infrared spectroscopy (NIRS), and transcranial doppler (TCD). The primary outcome of interest was the quantifiable effect that the induction of BS has on these measures of CBF. Animal studies included all studies performed in non-human animals including but not limited to rats, canines, swine, and non-human primates. Human studies included adults as well as pediatric populations.

Both continuous and intermittent methods of measures of CA were included in the search, a complete list of which can be found in Supplementary Appendix S1A.

Inclusion criteria for this study were as follows: BS measured by EEG in some form (visualized, bispectral index, power analysis); animal subjects, human participants, original full-length papers, use of some form of CBF measurement including the above, and/or a measurement of CA. Exclusion criteria included: non-English language and non-original studies.

### 2.2 Search strategy

MEDLINE, BIOSIS, Cochrane library, EMBASE, Global Health, and SCOPUS were all searched from their inceptions to August 2022 using relevant search terms. An example of the used search strategy can be found in Supplementary Appendix S1B. Search results were then compiled and deduplicated before being filtered for inclusion.

### 2.3 Study selection

A two-step review of all articles returned by our search was conducted using two reviewers (A.Z.S. and L.F.). In the first filtering phase, reviewers independently screened all titles and abstracts for possible inclusion. Next, full texts of articles that passed the first filtering phase were assessed to confirm that they met all inclusion criteria. Any discrepancies between the two reviewers were resolved by the senior author (F.A.Z.). Finally, the reference lists for each included article were screened for any missed articles.

### 2.4 Data collection

The following data was extracted from each of the final included articles and is presented in Supplementary Tables S1, S2: Study subject, experimental conditions, number of subjects, method of BS, method of BS determination, the measure of CBF, study results and conclusions, and study limitations.

### 2.5 Statistical analysis

Due to the heterogenous nature of the study designs and results of the relevant literature, no formal meta-analysis was conducted.

### 2.6 Bias assessment

Given the overall goal of this review, to perform a comprehensive systematic review of the literature, a formal bias assessment was not conducted.

## 3 Results

**FIGURE 2:**
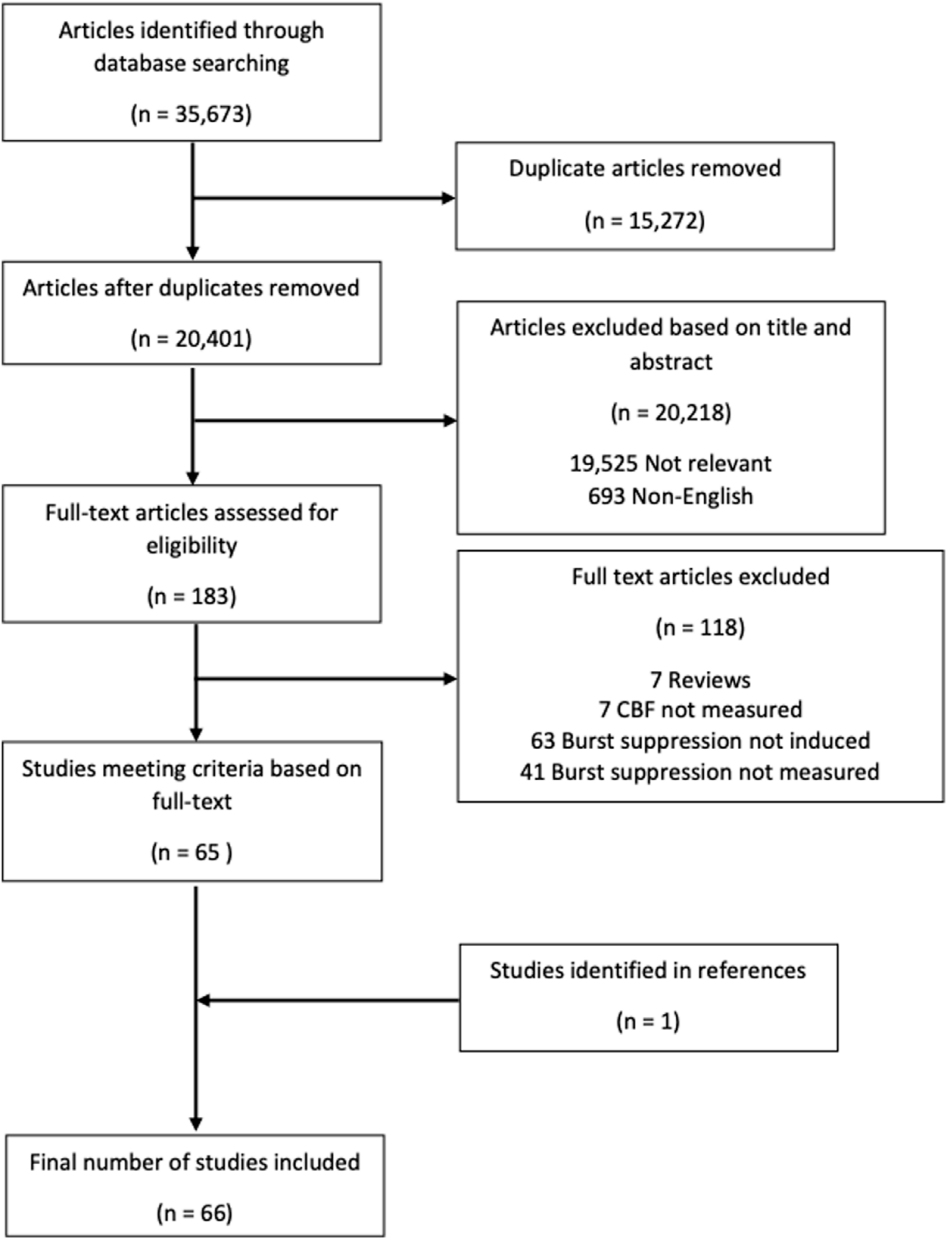
PRISMA flow diagram. PRISMA, preferred reporting items for systematic reviews and meta analyses.

The overall search and filtration results have been summarized in Figure 2 using a PRISMA flow-diagram. The total results of the search, which was carried out over all five databases, yielded 35,673 articles. After deduplication, 20,401 unique articles were identified. Based on their title and abstract, 20,218 articles were found to not meet the inclusion/exclusion criteria during the first filter phase. The full text of the remaining 183 articles was reviewed in the second filter phase. After reviewing the full text, 118 articles were found to not meet inclusion/exclusion criteria leaving 65 articles. Examination of the reference sections of those texts yielded one additional article resulting in 66 studies being included in this scoping review.

Overall, there were 66 studies that were included in the final results, 41 of which examined animals (Michenfelder, 1974; Harper and MacKenzie, 1977; Kassell et al., 1980; Gronert et al., 1981; Newberg et al., 1983; Milde et al., 1985; Maekawa et al., 1986; Scheller et al., 1986; Baughman et al., 1989; Warner et al., 1989; Lutz et al., 1990; Zornow et al., 1990; Roald et al., 1991; Artru et al., 1992; Ramani et al., 1992; Werner et al., 1992; Kochs et al., 1993; Golanov and Reis, 1995; Werner et al., 1995; Nemoto et al., 1996a; Nemoto et al., 1996b; Klementavicius et al., 1996; Young et al., 1997; Zarchin et al., 1998; Schmid-Elsaesser et al., 1999; Westermaier et al., 2000; Mäkiranta et al., 2002; Joshi et al., 2004; Joshi et al., 2005; Hungerhuber et al., 2006; Joshi et al., 2006; Liu et al., 2011; Liu et al., 2013a; Liu et al., 2013b; Walter et al., 2013; Sutin et al., 2014; Choi et al., 2016; Benveniste et al., 2017; Yang et al., 2018; Zhang et al., 2019; Berndt et al., 2021), 24 of which examined humans (Bendtsen et al., 1985; Woodcock et al., 1987; Young et al., 1991; Matta et al., 1995a; Matta et al., 1995b; Lam et al., 1995; Matta and Lam, 1995; Newman et al., 1995; Artru et al., 1997; Hoffman et al., 1997; Doyle and Matta, 1999; Matta et al., 1999; Ludbrook et al., 2002; Johnston et al., 2003; Reinsfelt et al., 2003; Reinsfelt et al., 2011; Zanatta et al., 2013; Du et al., 2014; Connolly et al., 2015; Chalia et al., 2016; Golkowski et al., 2017; Sahinovic et al., 2018; Akbik et al., 2020; Chaix et al., 2020; Kassab et al., 2021), and 1 of which examined both (Sirmpilatze et al., 2022). The study (Sirmpilatze et al., 2022) that examined both humans and animals will be included in both the *animal* and *human* sections. The animal studies are described in Supplementary Table S1 and the human studies are described in Supplementary Table S2. A brief overview of the studies can be seen in Table 1.

**TABLE 1:** Summary of the effect of different methods of BS on CBE and Autortgalation. GABA = a *γ-* aminobutyric acid; CMR_O2 =_ Cerebral metabolic rate for oxygen ; AVD_O2 =_ Arterio-venous different in oxygen.

Method of burst suppression	Mechanism of action	BS effect on CBF	Effect on Autoregulation	To Be determined
Propofol	GABA Agonist; readily crosses blood brain barrier (1)	In majority of studies analyzed, decrease was seen.Maximum drop was 70% (2–4) in animals and 44% (5) in humans. In animal studies, MAP maintenance was variable. In most human studies, MAP was maintained with pressors	Unclear; in human studies, five studies demonstrated a significant decrease in CBF (5–10) and all but one (9) showed no significant change in the AVD_O2_	Effect on brain-injured patients; mechanisms by which CBF is reduced other than flow-metabolic coupling
Barbiturates	GABA Agonist; Longer half-life than propofol	In majority of animal studies, significant reduction in CBE was seen (3,4,11–19). Maximum drop in animals was 80%. In two of four human studies, Reduction in CBF was seen to a maximum of 48%. In animal studies, MAP was either not maintained, or maintained with donor blood or pressers. In human studies, in one study MAP was maintained with pressors but in the others it is unclear	Unclear; Six animal studies showed a decrease in CMR_O2_ (11,13,15–17,20); five of these showed a decrease proportional to the decrease in CBF(11,15–17,20)	More human studies need to be performed; mechanisms by which CBF is reduced other than flow-metabolic coupling
Inhalational	Complex (21). Reduction in activation in the thalamus and midbrain reticular formations. GABA agonist; effects on other channels	In animal studies, variable effect, depending on brain region. Four animal studies showed decrease (22–25). Several studies demonstrated strong correlation between EEG	Unclear; high variability in effect on CBE in animal studies and AVD_O2_. Not examined in human studies	Effect on CBF; Effect on AVD_O2_ in humans

### 3.1 Animal studies

Of the 42 studies that examined animals, six used propofol (Artru et al., 1992; Ramani et al., 1992; Werner et al., 1992; Joshi et al., 2006; Wang et al., 2011; Liu et al., 2013a), 13 used barbiturates (thiopental) (Michenfelder, 1974; Kassell et al., 1980; Gronert et al., 1981; Milde et al., 1985; Nemoto et al., 1996a; Nemoto et al., 1996b; Klementavicius et al., 1996; Zarchin et al., 1998; Schmid-Elsaesser et al., 1999; Westermaier et al., 2000; Mäkiranta et al., 2002; Joshi et al., 2005; Hungerhuber et al., 2006), 18 used inhalational anaesthetics (isoflurane) (Newberg et al., 1983; Maekawa et al., 1986; Scheller et al., 1986; Baughman et al., 1989; Lutz et al., 1990; Zornow et al., 1990; Roald et al., 1991; Kochs et al., 1993; Golanov and Reis, 1995; Liu et al., 2011; Walter et al., 2013; Sutin et al., 2014; Choi et al., 2016; Benveniste et al., 2017; Yang et al., 2018; Zhang et al., 2019; Berndt et al., 2021; Sirmpilatze et al., 2022), four used a combination of methods (Harper and MacKenzie, 1977; Warner et al., 1989; Young et al., 1997; Joshi et al., 2004), and one induced BS through hypotension (Werner et al., 1995).

In terms of the subjects that were used in the studies, 12 used different species of dogs (beagle, mongrel) (Michenfelder, 1974; Kassell et al., 1980; Gronert et al., 1981; Newberg et al., 1983; Milde et al., 1985; Lutz et al., 1990; Zornow et al., 1990; Roald et al., 1991; Artru et al., 1992; Werner et al., 1992; Kochs et al., 1993; Werner et al., 1995), 18 used different species of rodents (Sprague Dawley rats, wistar rats, gerbils) (Maekawa et al., 1986; Baughman et al., 1989; Warner et al., 1989; Golanov and Reis, 1995; Nemoto et al., 1996a; Nemoto et al., 1996b; Klementavicius et al., 1996; Young et al., 1997; Zarchin et al., 1998; Schmid-Elsaesser et al., 1999; Westermaier et al., 2000; Hungerhuber et al., 2006; Liu et al., 2011; Liu et al., 2013a; Sutin et al., 2014; Choi et al., 2016; Benveniste et al., 2017; Berndt et al., 2021), six used different species of rabbits (Scheller et al., 1986; Ramani et al., 1992; Joshi et al., 2004; Joshi et al., 2005; Joshi et al., 2006; Wang et al., 2011), two used different species of swine (Mäkiranta et al., 2002; Walter et al., 2013), three used different species of non-human primates (Harper and MacKenzie, 1977; Yang et al., 2018; Zhang et al., 2019), and one used both rodents and non-human primates (Sirmpilatze et al., 2022).

#### 3.1.1 Propofol

Out of the six studies that used propofol to induce BS, two measured CBF by using the Laser Doppler Flowmetry Method (Joshi et al., 2006; Wang et al., 2011), one by measuring blood flow in the superior sagittal sinus (Artru et al., 1992), one by functional MRI and BOLD signal analysis (Liu et al., 2013a), one by hydrogen clearance method (Ramani et al., 1992), and one by both quantitative autoradiography and TCD (Werner et al., 1992). Overall, four of the studies showed a significant change in CBF with BS, all of them showing a significant decrease (Artru et al., 1992; Ramani et al., 1992; Werner et al., 1992; Joshi et al., 2006). The lowest decrease that was seen was 16% (Joshi et al., 2006) and the highest was 70% (Artru et al., 1992; Werner et al., 1992). Further, the authors found that there was a significant correlation between cortical CBF and CBF velocity (*r* = 0.86; *p* < 0.05), and that cortical cerebral vascular resistance (CVR) and PI were significantly correlated (*r* = 0.79; *p* < 0.05).

Overall, there was only one paper that was found that examined an established measure of CA (Werner et al., 1992). Werner et al., found that, when BS was achieved, CBF decreased by 70% and that this decrease was paralleled by a 210% increase in CVR and a 50% increase in the PI (Werner et al., 1992). While the other studies did not look at direct measures of CA, the effect on CA can be inferred from the effect of BS on MAP and CBF concurrently. In all of the studies that showed a significant change in CBF under BS, there was a disproportionately larger decrease in CBF compared with the MAP (Artru et al., 1992; Ramani et al., 1992; Werner et al., 1992; Joshi et al., 2006) and in one study, CBF fell by 70% while MAP remained unchanged (Werner et al., 1992). One study investigated CBF under normocapnia and hypocapnia finding similar decreases in CBF with normocapnia and BS (76%) and with hypocapnia and BS (77%) (Artru et al., 1992). However, interestingly, Wang et al. did not find a significant effect of BS on CBF (Wang et al., 2011). This may be due to the fact that rabbits were already sedated with propofol in the baseline condition, confounding the results although this would suggest CBF is related to sedation instead of BS. Liu et al. used fMRI BOLD signal analysis and found increased subcortical connectivity under BS but the implications on CBF remain unclear (Liu et al., 2013b).

Out of the propofol studies, two examined CMR_O2_ in addition to CBF (Artru et al., 1992; Werner et al., 1992). Artru et al., found a 30% significant reduction in CMR_O2_ to parallel the 70% decrease in CBF(21) and Werner et al., found a 70% decrease in CMR_O2_ to match the 70% decrease in CBF(24). In summary, most of the evidence suggests that BS induced by propofol reduces CBF, but it remains unclear if the decrease in CBF induced by BS is associated with a concurrent decrease in CMR_O2_. Overall, there is a lack of evidence regarding the effect of BS induced by propofol on CA as assessed by well-established methods of CA measurement. However, given that CBF is disproportionately affected compared with MAP, there is some evidence to suggest that CA impairs autoregulation and the Lassen Curve (Lassen, 1959; Ruesch et al., 2017).

#### 3.1.2 Barbiturates

Out of the 13 studies that used barbiturates to induce BS, five measured CBF using the Laser Doppler Flowmetry Method (Zarchin et al., 1998; Schmid-Elsaesser et al., 1999; Westermaier et al., 2000; Joshi et al., 2005; Hungerhuber et al., 2006), three by measuring blood flow in the superior sagittal sinus (Michenfelder, 1974; Gronert et al., 1981; Milde et al., 1985), one by functional MRI and BOLD signal analysis (Mäkiranta et al., 2002), three by hydrogen clearance method (Nemoto et al., 1996a; Nemoto et al., 1996b; Klementavicius et al., 1996), one by quantitative autoradiography (Kassell et al., 1980). All of the studies used thiopental except for one that used pentobarbital (Gronert et al., 1981), one that used methohexital (Westermaier et al., 2000), and one that used etomidate (Milde et al., 1985). While etomidate is not technically a barbiturate, it is considered in this section as it is more like the barbiturate medications compared with propofol or inhalational anaesthetics.

Overall, 11 studies showed a significant reduction in CBF with induction of BS(25–28,30,33–37,87). The amount of reduction in CBF ranged from 23% (Zarchin et al., 1998) to 80% (Hungerhuber et al., 2006) in the studies in which the values are reported. Three of the studies did not measure or report change in MAP (Zarchin et al., 1998; Schmid-Elsaesser et al., 1999; Hungerhuber et al., 2006). Similar to the pattern seen in the propofol studies, in all of the studies in which MAP was recorded (Michenfelder, 1974; Kassell et al., 1980; Gronert et al., 1981; Milde et al., 1985; Nemoto et al., 1996a; Nemoto et al., 1996b; Klementavicius et al., 1996; Westermaier et al., 2000), the decrease in CBF was disproportionately greater than the decrease in MAP. Westermaier et al., showed that there was a 30% greater reduction in CBF in hypothermic rats that were burst suppressed compared with normothermic rats that were burst suppressed (*p* < 0.05) (Westermaier et al., 2000). However, this was not seen in another study where BS caused an equal 50% reduction in CBF in the normothermic and hypothermic rats (Nemoto et al., 1996a).

Out of the seven studies that examined CMR_O2_ (Michenfelder, 1974; Kassell et al., 1980; Gronert et al., 1981; Milde et al., 1985; Nemoto et al., 1996a; Nemoto et al., 1996b; Klementavicius et al., 1996), six showed a decrease that ranged from 42% to 54% (Michenfelder, 1974; Kassell et al., 1980; Milde et al., 1985; Nemoto et al., 1996a; Nemoto et al., 1996b; Klementavicius et al., 1996). The remaining study used pentobarbital and showed a 25% increase in CMR_O2_ in canines (Gronert et al., 1981). One noteworthy study found a strong positive correlation between CBF and CMR_O2_ (*R* = 0.77; *p* < 0.05) and a strong negative correlation between CVR and CMR_O2_ (*R* = −0.77; *p* < 0.05) (Kassell et al., 1980). One notable study demonstrated that CVR was inversely related to CBF, increasing to 214% of baseline when CBF was at 50% of baseline, and increasing to 300% of baseline when CBF reached its trough (Milde et al., 1985).

There were two studies that did not demonstrate a reduction in CBF with induction of BS (Mäkiranta et al., 2002; Joshi et al., 2005). One used fMRI and demonstrated that there was an increase as well as decrease in the BOLD signal depending on the specific region of interest in pigs (Mäkiranta et al., 2002). Joshi et al. measured the effect that BS induced by thiopental has on CBF using laser doppler flowmetry in rabbits but did not find any significant change (Joshi et al., 2005).

In summary, like the propofol studies, almost all of the studies demonstrated that BS causes a significant reduction in CBF. The reduction in CBF is paralleled by a decrease in CMR_O2_ implying that it may be due to reduced brain metabolism. Overall, there is a lack of evidence regarding the effect of BS induced by propofol on CA as assessed by well-established methods of CA measurement. However, again given the connection between CBF and CA, there may be some impairment of autoregulation.

#### 3.1.3 Inhalational

Out of the 18 studies that used inhalational anaesthetics to induce BS, four measured blood flow in the superior sagittal sinus (Newberg et al., 1983; Lutz et al., 1990; Zornow et al., 1990; Roald et al., 1991), two used the Laser Doppler Flowmetry Method (Golanov and Reis, 1995; Berndt et al., 2021), two used quantitative autoradiography (Maekawa et al., 1986; Baughman et al., 1989), three used near infrared spectroscopy (Sutin et al., 2014; Choi et al., 2016; Yang et al., 2018), two used functional MRI and BOLD signal analysis (Zhang et al., 2019; Sirmpilatze et al., 2022), two used FDG-PET (Walter et al., 2013), two used hydrogen clearance method (Scheller et al., 1986), one analyzed glymphatic transport and used magnetic resonance time of flight (Liu et al., 2013b), one used laser doppler flowmetry and fMRI (Liu et al., 2011), and one used quantitative autoradiography and TCD (Kochs et al., 1993).

Almost all of the studies that used inhalational anaesthetic used isoflurane except for one that used desflurane (Lutz et al., 1990) and one that used nitrous oxide in addition to isoflurane (Baughman et al., 1989). There was more variability in the results of the isoflurane studies which may be in part be due to the variability in the techniques used. Of the studies that looked at overall CBF, four studies showed that BS led to a decrease in CBF (Baughman et al., 1989; Zornow et al., 1990; Walter et al., 2013; Berndt et al., 2021), two showed an increase in CBF (Ramani et al., 1992; Gomez et al., 2022), three showed no change (Newberg et al., 1983; Scheller et al., 1986; Roald et al., 1991), and two showed an increase or decrease depending on the region of the brain and the concentration of anaesthetic used (Maekawa et al., 1986; Lutz et al., 1990). In the remainder, conclusions could not be drawn on the average effect of BS on CBF but did shed light on neurovascular coupling (Golanov and Reis, 1995; Liu et al., 2011; Sutin et al., 2014; Zhang et al., 2019; Sirmpilatze et al., 2022). For example, several studies showed strong coupling between BS spikes and spikes in CBF (Golanov and Reis, 1995; Liu et al., 2011; Sutin et al., 2014; Zhang et al., 2019; Berndt et al., 2021). In one notable study that used FDG-PET, during BS there was a 48% reduction in CBF post onset of BS which was paralleled by a 42% reduction in CMR_O2_ (Walter et al., 2013). Further, there was a significant 31% reduction of metabolism for glucose in the neocortex, 25% in the basal ganglia, 20% reduction in the thalamus in neonatal pigs lending support to the concept that BS reduces CBF by reducing cerebral metabolic demand. In another study that used near infrared spectroscopy, as the duration of BS increased, total hemoglobin (Hgb) (*R* ^2^ = 0.53, *p* < 0.05), and oxygenated hemoglobin (oxyHgb) significantly increased (*R* ^2^ = 0.72, *p* < 0.05), and the amount of deoxygenated hemoglobin (deoxyHgb) significantly decreased (*R* ^2^ = −0.92; *p* < 0.05) (Choi et al., 2016).

In summary, there still exists heterogeneity in the literature regarding the effects that BS induced by inhalational anaesthetics has on CBF and autoregulation. However, there is evidence to suggest that there is tight neurovascular coupling in the brain and spikes on EEG during the BS state are associated with concurrent spikes in CBF. Like the other anaesthetics, there is a paucity of literature on the effect of BS on CA measures.

#### 3.1.4 Multiple and other

Four studies used multiple methods to induce BS. Two used a barbiturate and inhalational anaesthetic (Harper and MacKenzie, 1977; Warner et al., 1989) but in one (Harper and MacKenzie, 1977), there was no baseline measurement of CBF prior to induction of BS so the conclusions that can be drawn are limited. In the other (Warner et al., 1989), compared to the sham condition, there was 67% lower CBF in the pentobarbital condition but no difference in the CBF in the isoflurane condition. Interestingly, there was no difference in MAP or in CMR_glu_ with pentobarbital or isoflurane. Another study used a barbiturate and propofol (Joshi et al., 2004) to induce BS in rabbits but neither induced a significant change in CBF. Finally, one used an inhalational anaesthetic and propofol (Young et al., 1997) in an ischemia reperfusion model in rats and while CBF was not directly measured, the infarct volume in the propofol group was 21% significantly lower than in the isoflurane group (*p* < 0.001). This may suggest greater decrease in cerebral metabolism with propofol compared with isoflurane.

One study also used hemorrhagic hypotension to induce BS in dogs (Werner et al., 1995) but the experimental design itself examined the effect of CBF on BS instead of *vice versa*, limiting the conclusions that can be drawn.

In summary, there is mild evidence to suggest that barbiturates and propofol may have stronger effects on CBF compared with inhalational anaesthetics, but more studies are needed comparing the different anaesthetics. The effect on CA remains unclear.

### 3.2 Human studies

Of the 24 studies that examined humans, six used propofol (Matta et al., 1995a; Newman et al., 1995; Doyle and Matta, 1999; Ludbrook et al., 2002; Klein et al., 2011; Chaix et al., 2020), four used barbiturates (thiopental) (Bendtsen et al., 1985; Young et al., 1991; Hoffman et al., 1997; Connolly et al., 2015), six used inhalational anaesthetics (isoflurane) (Lam et al., 1995; Artru et al., 1997; Reinsfelt et al., 2003; Reinsfelt et al., 2011; Golkowski et al., 2017; Sirmpilatze et al., 2022), six used a combination of methods (Woodcock et al., 1987; Matta et al., 1995b; Matta et al., 1999; Zanatta et al., 2013; Akbik et al., 2020), and three looked at the effect of brain injury on BS (Du et al., 2014; Chalia et al., 2016; Kassab et al., 2021).

In terms of the participants that were used in the studies, five involved adults undergoing cardiac surgery (coronary bypass graft or valve) (Woodcock et al., 1987; Newman et al., 1995; Reinsfelt et al., 2003; Reinsfelt et al., 2011; Zanatta et al., 2013), five involved adults undergoing open neurosurgical procedures (Bendtsen et al., 1985; Artru et al., 1997; Hoffman et al., 1997; Doyle and Matta, 1999; Klein et al., 2011), six involved adults undergoing orthopedic and other surgeries (Matta et al., 1995a; Matta et al., 1995b; Lam et al., 1995; Matta and Lam, 1995; Matta et al., 1999; Ludbrook et al., 2002), one involved patients undergoing carotid endarterectomy (Young et al., 1991), one involved patients undergoing minimally invasive neuroradiological procedures (Chaix et al., 2020), five involved critically ill and/or brain injured patients (Johnston et al., 2003; Connolly et al., 2015; Sahinovic et al., 2018; Akbik et al., 2020; Kassab et al., 2021), one involved infant patients with hypoxic ischemic encephalopathy (Chalia et al., 2016), and two involved healthy adults (Golkowski et al., 2017; Sirmpilatze et al., 2022).

#### 3.2.1 Propofol

Out of the six studies that used propofol to induce BS, one measured CBF using the Laser Doppler Flowmetry Method (Du et al., 2014), one by the Xe clearance (Newman et al., 1995), three by TCD (Lam et al., 1995; Doyle and Matta, 1999; Ludbrook et al., 2002), one by TCD and NIRS (Chaix et al., 2020).

Out of the six studies that used propofol to induce BS, five directly looked at CBF and all of them found a significant decrease in CBF (Matta et al., 1995a; Newman et al., 1995; Doyle and Matta, 1999; Ludbrook et al., 2002; Chaix et al., 2020). The highest reduction seen in CBF velocity was 42% (*p* < 0.0001) and it was achieved in adult patients undergoing orthopedic procedures (Ludbrook et al., 2002) and the highest decrease in CBF was 44% (*p* < 0.05), seen in adult patients undergoing cardiac surgery (note that in this study, the CBF was compared between groups rather than within groups) (Newman et al., 1995). In one notable study which looked at BS in addition to hypothermia, CBF was significantly lower in burst suppressed patients compared to controls at normothermia (44%; *p* < 0.05), hypothermia (41%; *p* < 0.05), and during rewarming (37%; *p* < 0.05); CMR_O2_ followed the same pattern (Newman et al., 1995). In all the studies, MAP either remained the same or did not decrease as much as CBF. In another study, patients receiving propofol were divided into two groups: one that included patients with more cardiovascular risk factors (“high risk”) and another with fewer cardiovascular risk factors (“low risk”) (Chaix et al., 2020). The “high risk” group had a higher drop in CBF with BS (34%) compared with the “low risk patients” (17%; *p* < 0.001) suggesting that patients with risk factors may already have altered CA. Overall, there is a lack of evidence regarding the effect of BS induced by propofol on CA as assessed by well-established methods of CA measurement.

In the one remaining study, venous capillary blood flow was measured *in lieu* of arterial CBF and there was no significant change found (Klein et al., 2011).

There were four studies that examined AVD_O2_ (Newman et al., 1995; Doyle and Matta, 1999; Ludbrook et al., 2002; Klein et al., 2011); all of the studies found no significant change in AVD_O2_ except for one which examined patients undergoing elective craniotomy and found that there was a 25% significant decrease and 58% significant decrease in AVD_O2_ in the group with lower bispectral index compared with the group with higher bispectral index (*p* = 0.022) (Klein et al., 2011).

#### 3.2.2 Barbiturates

Out of the 4 studies that used barbiturates, one uses althesin (Bendtsen et al., 1985), one used pentobarbital (Connolly et al., 2015), one used thiopental (Young et al., 1991), and one used etomidate (Hoffman et al., 1997).

In two of the studies, BS led to a change in CBF (Young et al., 1991; Hoffman et al., 1997); in Hoffman et al., there was a significant decrease of 40% in CBF (*p* < 0.05) which was also accompanied by a decrease in CMR_O2_ and in Young et al., there was a 48% significant decrease in CBF (*p* < 0.01). In the former, there was a 10% decrease in MAP and in the latter, phenylephrine was used to maintain MAP. In the other two studies, CBF was either not measured at baseline without BS, limiting the conclusions that can be drawn (Bendtsen et al., 1985) or the sample size was extremely small at 2 participants (Connolly et al., 2015). This evidence suggests that barbiturates decrease CBF and may impact CA but there is a dearth of literature regarding barbiturates compared with well-established measures of CA.

#### 3.2.3 Inhalational

Out of the six studies that used inhalational anaesthetics, three used sevoflurane (Reinsfelt et al., 2011; Golkowski et al., 2017; Sirmpilatze et al., 2022), one used isoflurane (Reinsfelt et al., 2003), and the others used a combination of sevoflurane, isoflurane, and/or desflurane (Lam et al., 1995; Artru et al., 1997). There were four studies that showed a consistent effect on CBF or CBF velocity (Lam et al., 1995; Artru et al., 1997; Reinsfelt et al., 2003; Reinsfelt et al., 2011), all of which showed a consistent decrease after induction of BS. Notably, Artru et al, (1997) showed a 30% reduction in CBF velocity accompanied by a 36% reduction in CPP (*p* < 0.05) but no change in CVR with isoflurane, and 32% reduction in CBF velocity and 41% increase in CVR (*p* < 0.05) without a significant reduction in CPP with sevoflurane. There was only a 10% reduction in MAP in the isoflurane group and no reduction in MAP in the sevoflurane group. Reinsfelt et al, (2011) showed that BS led to a 27% reduction in CBF velocity (*p* < 0.05) but more interestingly, that this was also accompanied by a 13% decrease in cerebral oxygen extraction (COE) (*p* < 0.05). Further, under BS, there was a steeper positive slope of relationship between CBF velocity and CPP and a steeper negative slope between CPP and COE (*p* < 0.05), which may suggest the effect of sevoflurane was through vasodilation.

The remaining two studies performed BOLD signal analysis and showed that there was more widespread fluctuation in BOLD signal and significant correlation with cortical sensory areas and the thalamus during BS (*p* < 0.05) (Sirmpilatze et al., 2022) or a lower amplitude of the BOLD signal and significant correlation of the BOLD signal with BS in frontal, parietal, and temporal lobes and in basal ganglia (*p* < 0.001) (Golkowski et al., 2017).

In summary, inhalational anaesthetics may reduce CBF but more evidence is needed to determine what effect that these anaesthetics have on CVR and cerebral metabolism. Further, much like the studies with the other anaesthetics, there is a lack of evidence regarding the evidence regarding BS induced by inhalational anaesthetics and CA.

#### 3.2.4 Multiple

There were four studies that investigated the use of propofol in addition to inhalational anaesthetics (Matta et al., 1995b; Matta and Lam, 1995; Matta et al., 1999; Zanatta et al., 2013). Interestingly, all the studies showed that when sevoflurane, isoflurane, halothane, desflurane or nitric oxide are added to propofol, there is a corresponding increase in CBF velocity. There is also evidence to suggest that when nitric oxide is added to propofol, there is a corresponding 16% decrease in CVR and 14% increase in CMR_O2_ (*p* < 0.05) (Matta and Lam, 1995).

Woodcock et al. (1987) compared the effect that thiopental has on CBF with the effect of isoflurane under normothermia and hypothermia. Under both conditions, CBF was significantly lower in the thiopental burst suppressed group compared to control but there was no significant difference in CBF between isoflurane and control. Surprisingly, in the normothermic condition, CMR_O2_ was 21% lower in the thiopental group and 34% lower in the isoflurane group compared to control (*p* < 0.05). This difference was 34% and 29% respectively for thiopental and isoflurane in the hypothermic condition (*p* < 0.05). Taken together, this may mean that inhalational anaesthetics, while having a significant effect on cerebral metabolic demand, do not have as significant effect on CBF and autoregulation but more comparative studies are needed.

Finally, one study used FDG-PET with a smaller and more heterogenous sample size did not look at CBF directly (Akbik et al., 2020). However, it did show that BS led to reduced FDG uptake in patients with status epilepticus, stroke-like migraine attacks after radiotherapy, and epilepsy secondary to cerebral amyloid angiopathy but led to increased FDG uptake in patients with viral or autoimmune encephalitis. This may reflect the effect that underlying disease processes have on cerebral metabolism and the subsequent consequences that anaesthesia can have.

Overall, more research is needed regarding the differential effects of anaesthetics on CBF as well as how different anesthetics effect CA.

#### 3.2.5 Brain injury

There were three studies that investigated the effect that brain injury has on EEG, CBF, and neurovascular coupling (Chalia et al., 2016; Sahinovic et al., 2018; Kassab et al., 2021). Kassab and others showed that, in a group of patients in status epilepticus, there was a significant positive correlation between seizure duration on EEG and hemodynamic response function (oxygenated hemoglobin *p* = 0.21 *R* ^2^ = 0.569, total hemoglobin *p* = 0.034 *R* ^2^ = 0.403); when BS was induce there was an increase in oxygenated hemoglobin and total hemoglobin following onset of bursts in representative hemodynamic response functions (Kassab et al., 2021). Chalia and others showed that there was a pronounced decrease in oxygenated hemoglobin just before or during EEG burst activity followed by large increase reaching peak 20s after burst onset (Chalia et al., 2016). Taken together, these studies suggest tight neurovascular coupling during BS.

## 4 Discussion

### 4.1 Overview

In this systematically conducted scoping review, we identified 66 studies, most of which involved animals. This should be expected as manipulation of CBF using anaesthetics poses significant risk to humans and can only be investigated in participants who are receiving anaesthetics for some other purpose. This is also why most human studies involved patients who were injured or receiving a therapeutic surgery.

By far, most of the studies involved small animal models (rodents and rabbits), which are not as applicable to humans and is a weakness of the literature. The largest sample sized used in the small-animal models was 61 (Maekawa et al., 1986) and involved Sprague-Dawley rats. The largest sample size seen in the large animal models was 16 and involved non-human primates, but only two of the primates achieved BS (Zhang et al., 2019).

Six animal and seven human studies investigated propofol. Propofol is a *γ*-aminobutyric acid (GABA) agonist with a rapid terminal half life that readily crosses the blood brain barrier (Sahinovic et al., 2018). The most common adverse effects are bradycardia, hypotension, and very rarely, the life-threatening propofol infusion syndrome. In both animals and humans, propofol consistently showed a decrease in CBF in most studies of up to 70% in animals and up to a 44% decrease in humans. There were only two animal studies amongst the studies that showed a change in CBF that examined CMR_O2_ (Artru et al., 1992; Werner et al., 1992). One found that the CBF decreased to the same proportion as the CMR_O2_ (Werner et al., 1992) and another found that the CBF decreased disproportionately much greater compared to the decrease in CMR_O2_ (Artru et al., 1992). The former result would be expected if the decrease in CBF was purely due to flow-metabolism coupling whereas the latter would imply that propofol may have also had direct effects on the smooth muscle of the cerebral blood vessels themselves in addition to causing a reduction in CMR. Interestingly, in the human studies, while five studies demonstrated a significant decrease in CBF (Matta et al., 1995a; Newman et al., 1995; Doyle and Matta, 1999; Ludbrook et al., 2002; Klein et al., 2011; Chaix et al., 2020), all but one (Klein et al., 2011) showed no significant change in the AVD_O2,_ supporting the conclusion that propofol decreases CBF through mechanisms other than flow-metabolism coupling. In the one study that investigated patients with TBI, even though CBF was not measured, there was no significant effect found on ICP or AVD_O2_ (Johnston et al., 2003). Interestingly, in one animal study, propofol reduced CBF by similar amounts under both normocapnia and hypocapnia (Artru et al., 1992) and in one human study, decreased CBF by similar amounts under both hypothermia and normothermia (Newman et al., 1995). Both hypothermia and hypercapnia are known to reduce CBF through vasoconstriction (Tameem and Krovvidi, 2013) and interestingly, it seems that when propofol is added, despite the fact that it reduces CBF on its own, the effect does not seem to be synergistic. Interestingly, it seems that patients who have cardiovascular risk factors have a higher reduction in CBF compared with those who have fewer risk factors (Chaix et al., 2020). In patients with hypertension, due to factors such as arterial remodelling and renin-angiotensin system, the CA curve is shifted to the right and cerebral ischemia can occur at higher MAPs compared to patients without hypertension (Tameem and Krovvidi, 2013; Machado et al., 2021).

Barbiturates have a similar mechanism of action to propofol but have longer half-lives and have higher risk of inducing hypotension (Shin et al., 2018). The number of animal studies that investigated barbiturates was comparable to the other anaesthetics; this contrasted with the human studies of which only four investigated barbiturates. Eleven of the animal students demonstrated that barbiturates had a significant reduction in CBF to a maximum of 80% (Michenfelder, 1974; Kassell et al., 1980; Gronert et al., 1981; Milde et al., 1985; Nemoto et al., 1996a; Nemoto et al., 1996b; Klementavicius et al., 1996; Zarchin et al., 1998; Schmid-Elsaesser et al., 1999; Westermaier et al., 2000; Hungerhuber et al., 2006). Six animal studies showed a decrease in CMR_O2_ (Michenfelder, 1974; Kassell et al., 1980; Milde et al., 1985; Nemoto et al., 1996a; Nemoto et al., 1996b; Klementavicius et al., 1996) and out of those six studies, five showed a decrease proportional to the decrease in CBF (Michenfelder, 1974; Kassell et al., 1980; Nemoto et al., 1996a; Nemoto et al., 1996b; Klementavicius et al., 1996). One study even found a strong positive correlation between CBF and CMR_O2_ (*R* = 0.77; *p* < 0.05) (Kassell et al., 1980). In another study in which CBF decreased disproportionately more than CMR_O2_ (75% vs. 54%), the anaesthetic used to induce BS was etomidate and CBF plateaued before BS was even reached (Milde et al., 1985). As mentioned earlier, etomidate is not technically a barbiturate and is structurally unrelated to other anaesthetics (Williams et al., 2022). Etomidate has been shown to cause depression in certain parts of the brain and cause hyperexcitability in others such as the basal ganglia. However, in the one human study that used etomidate for anaesthesia, the CBF and CMR_O2_ decreased proportionally by 40% (Hoffman et al., 1997). Therefore, it remains unclear that is the effect etomidate has on CBF is due to its decrease in CMR alone or also due to other mechanisms. Interestingly, the only animal study in which CMR_O2_ was increased used pentobarbital (Gronert et al., 1981). This was presumably due to tolerance as there is evidence to suggest that tolerance develops much quicker with pentobarbital compared to the other barbarities (Saunders et al., 1990). There was a significant lack of human studies that investigated barbiturates; out of the four, one used etomidate (Hoffman et al., 1997) and one only had a sample size of 2 (Connolly et al., 2015). The lack of studies investigating barbiturates on humans may be due to its unpopularity as an anaesthetic given their tendency to cause hypotension (Shin et al., 2018).

It has been well-described that inhalational anaesthetics produce deep sedation by reducing activation in the thalamus and midbrain reticular formations (Campagna et al., 2003). However, while there is a reduction in brain activation in several cortical areas, activation of the visual, motor, and subcortical regions seems to be unaffected. While inhalational anaesthetics primarily target the GABA receptor, they have effects on a plethora of other channels including glycine receptors, nicotinic acetylcholine receptors, and glutamate receptors (Campagna et al., 2003). There were a total of 18 animal and 6 human studies that used inhalational anaesthetics. The animal literature was extremely variable in terms of the effect that inhalational anaesthetics have on CBF and only four showed a decrease in CBF (Baughman et al., 1989; Zornow et al., 1990; Walter et al., 2013; Berndt et al., 2021). This may have been because, as previously mentioned, inhalational anaesthetics have different effects on the activity of different parts of the brain. In addition, the studies used a variety of concentrations of isoflurane, ranging from 1%–3%. In one study, at a concentration of 1.0 minimum alveolar concentration (MAC), there was no change in CBF but at 1.5 MAC the auditory cortex saw a 4.9% significant decrease in CBF (*p* < 0.05), the visual cortex did not see any significant change, and the extrapyramidal system (140%–170%) and limbic system (87%–120%) saw a significant increase, and finally, with 2.0 MAC, all regions saw a significant increase in CBF (Maekawa et al., 1986). Further, in one fMRI study, there was more widespread fluctuation in BOLD signal and significant correlation with cortical sensory areas and the thalamus during BS (Sirmpilatze et al., 2022) and in another a lower amplitude of the BOLD signal and significant correlation of the BOLD signal with BS in frontal, parietal, and temporal lobes and in basal ganglia (Golkowski et al., 2017). While CMR_glu_ decreased across the brain, it did not decrease in the hippocampus or corpus callosum at any concentrations (Maekawa et al., 1986). Several of the animal inhalational anaesthetic studies (Golanov and Reis, 1995; Liu et al., 2011; Sutin et al., 2014; Zhang et al., 2019; Berndt et al., 2021) demonstrated strong correlation between EEG bursts/spikes and spikes in CBF, lending support to the mechanisms behind flow-metabolism coupling in the control of CBF. Out of the six human studies that used inhalational anaesthetics, four showed a consistent decrease in CBF or CBF velocity (Roald et al., 1991; Kochs et al., 1993; Liu et al., 2013b; Yang et al., 2018). While sevoflurane did reduce CBF velocity and CBF (Artru et al., 1997; Reinsfelt et al., 2011), but it did not show any significant reduction in CPP (Artru et al., 1997). This was in contrast to isoflurane which showed a 36% reduction in CPP in the same study (Artru et al., 1997). None of the human studies examined the effect of the anaesthetics on CMR_O2_ or AVD_O2_ and therefore, many questions remain unanswered regarding the effect that isoflurane and other inhalational drugs have on CBF and to what extent the effect is mediated by a change in CMR.

In terms of the studies that investigated multiple anaesthetics, there was evidence to suggest that when inhalational anaesthetics are added to propofol, there is an increase in CBF velocity compared to the use of propofol alone. This may be because inhalational anaesthetics increase CBF in certain parts of the brain, thereby decreasing the effect that propofol has. Further, inhalational anaesthetics affect a variety of receptor sites including glutamate receptors and by doing so may nullify the effect of propofol (e.g., inhibition of inhibition). Compared to inhalational anaesthetics, barbiturates seem to have a higher effect on reducing CBF, which was seen in an animal (Warner et al., 1989) as well as a human study (Woodcock et al., 1987). This may be due in part to the higher likelihood of barbiturates to cause hypotension but may also be because inhalational anaesthetics have different effects depending on the region in the brain (Campagna et al., 2003; Golkowski et al., 2017; Sirmpilatze et al., 2022).

Overall, there was a significant lack of discussion of autoregulation in the studies that were examined in the present review, as will be discussed in the limitations section. Previous work has shown that, at lower doses, propofol, barbiturates, and inhalational anaesthetics do not affect CBF and autoregulation (Veselis et al., 2005; Farag et al., 2017; Slupe and Kirsch, 2018) at low doses but at higher doses CBF and autoregulation are impaired (Slupe and Kirsch, 2018). It was unclear why higher doses of anaesthetics cause this change. Given that most of the evidence pointed toward BS causing a reduction in CBF that was disproportionately greater than the reduction in MAP (Michenfelder, 1974; Kassell et al., 1980; Gronert et al., 1981; Milde et al., 1985; Artru et al., 1992; Ramani et al., 1992; Werner et al., 1992; Matta et al., 1995b; Newman et al., 1995; Nemoto et al., 1996a; Doyle and Matta, 1999; Westermaier et al., 2000; Ludbrook et al., 2002; Joshi et al., 2006; Chaix et al., 2020), it can possibly be inferred that BS has at least some effect on autoregulation impairment. While there was some evidence that CBF changes in proportion to CMR_O2_ (Michenfelder, 1974; Kassell et al., 1980; Milde et al., 1985; Werner et al., 1992; Nemoto et al., 1996a; Klementavicius et al., 1996), there was also evidence that CBF changed disproportionately (Milde et al., 1985; Baughman et al., 1989; Artru et al., 1992), making it challenging to know what effect exactly BS has on autoregulation. It is also possible that different depths of sedation have a significant effect on animal and human cerebral autoregulation. For example, previous work looked at the relationship between objectively measured depth of sedation (through the bispectrality index) and cerebrovascular reactivity (surrogate measure of cerebral autoregulation) in patients with TBI (Froese et al., 2022a; Froese et al., 2022b). From this work it was seen in almost all patients that there was a depth of sedation that optimized cerebrovascular reactivity (achieve the most intact cerebral reactivity value), indicating that too much/little sedation results in non-optimal cerebral states.

### 4.2 Limitations of the literature

In most of the studies, it was clear that BS decreases CBF, and this effect is most pronounced with propofol and barbiturates. However, it remains unclear why BS has this effect. CBF is believed to be controlled by four primary mechanisms: myogenic, endothelial, neurogenic, and flow-metabolism coupling (Tameem and Krovvidi, 2013). In the myogenic mechanisms, the smooth muscle cells themselves are responsible to modulating cerebral vessel diameter. The endothelial theory suggests that shear-stress induced by blood flow changes on the endothelial lining leading to induction of vasoactive mediators, such as nitric oxide or endothelin, precipitating changes in vascular tone. In the neurogenic mechanism, vasoconstriction is caused by norepinephrine and neuropeptide Y released from postganglionic sympathetic from the superior cervical ganglion. Finally, in flow-metabolic coupling, CBF is closely matched to the demands of the neuronal firing. Flow-metabolic coupling is thought to be mediated by astrocytes, a type of glial cell in the brain. While the process is not entirely understood, it is believed that astrocytes communicate the activity of neurons to blood vessels and coordinate the energy demand with oxygen and glucose supply (Venkat et al., 2016). Astrocytes may mediate the synthesis of vasoactive substances, thereby inducing vasodilation when neuronal activity increases. Reasonably, it is unlikely that the anaesthetics have much of an effect on the smooth muscle cells or on the sympathetic ganglion as much as they do on the neuronal activity. However, given that many studies did not investigate CMR_O2_ and a significant few looked at CMR_glu_, it is challenging to know if this is indeed the mechanism. One of the main confounders in the human studies was the maintenance of MAP. In some of the studies, MAP was maintained with pressors above a given level with pressors while in others, it was allowed to drop. First, if MAP is allowed to drop, it is difficult to state whether CBF dropped due to a drop in systemic hemodynamics or due to the BS induced by the anaesthetic. Interestingly, in previous papers our team has shown that, while pressors cause vasoconstriction when applied to cerebral blood vessels directly, there is no effect when applied systemically (Froese et al., 2020b; Froese et al., 2020c). Therefore, vasopressors do not appear to have a significant effect on CBF or autoregulation. Without MAP being maintained throughout the experiment, it is challenging to know if CBF dropped due to alteration of cerebral hemodynamics and therefore a myogenic mechanism or due to a flow-metabolic coupling method. Further, without estimating the amount of sedation that is given to the subjects, no firm conclusions can be drawn in relation to the effects that BS induced by anaesthetics has on CBF. More animal but especially more human studies are needed to clarify how and why BS causes a decrease in CBF.

It remains unclear what effect inhalational anaesthetics have on CBF as there were mixed results from the studies. These anaesthetics are especially interesting as they seem to not have as much hemodynamic depression (Campagna et al., 2003) as other agents and are especially useful for disease processes such as refractory status epilepticus (Mirsattari et al., 2004). Further, if inhalational anaesthetics are used in combination with other anaesthetics in the neurocritical care unit, could the profiles of both agents be used in a synergistic fashion? For example, adding isoflurane to propofol in a patient in whom it would be beneficial to reduce the CMR but not the CBF as much. There still exists a gap in the literature regarding inhalational anaesthetics as well as the use of a combination of anaesthetics in patients. There was a significant lack of studies that investigated the effect of BS on CBF in patients with critical neurological illness or traumatic brain injury. Given that these patients may have altered autoregulation (Armstead, 2016), it would be pertinent to see how these patients respond to the different anaesthetics.

Finally, most of the studies that were examined in the present review did not measure the effect of BS on autoregulation. From the results of the present review, we can conclude that BS leads to a decrease in CBF but given that autoregulation links CBF and CMR_O2_ by flow-metabolism coupling, the drop in CBF may be an *appropriate* response of autoregulation (Tameem and Krovvidi, 2013). There are two overarching methods of measuring autoregulation: a “static” method and a “dynamic” method (Tiecks et al., 1995). The older, static method involves measuring CBF at a constant baseline MAP and constant CBF. After manipulation of the MAP is undertaken, another measure of CBF is taken and if this measure changes significantly, there is an impairment in CA (Tiecks et al., 1995). The major disadvantage with this method is that there is no assessment of the time in which the change in CVR occurs. The dynamic method involves using a thigh blood pressure cuff (Obrist et al., 1975); the cuff is inflated and rapidly released which causes rapid drops in MAP. During this process, MAP and CBF velocity, measured using TCD, and the latency of the change in CVR can be measured. In using both methods with BS, there exists a confound. One possible method of inferring a loss of autoregulation is if CBF decreases disproportionately more than CMR_O2_ as they should otherwise be tightly coupled. More studies are needed to measure the effect of BS on CBF and CMR_O2_ using both static and dynamic methods.

### 4.3 Limitations of this review and future directions

One of the main limitations to the present review was its breadth. By design, the review’s purpose was to examine the effect of BS on overall cerebral physiology, hence, we were not selective about the methods used to measure CBF or to induce BS. The advantage to this was that we were able to assess the effect of BS in a wide variety of scenarios and in different species. However, by including a vast array of methodologies, we compromised on accuracy. For example, we included studies that used TCD in the present review even though TCD measures CBF velocity which is not completely analogous to CBF and under conditions in which the vessel diameter is variable, can under or overestimate CBF (Coverdale et al., 2014). In addition, we included studies that measured CBF using the BOLD signal, which only measures changes in oxygenated blood (Logothetis, 2002). Nevertheless, inclusion of these studies allowed us to examine the effect of BS on cerebral physiology in a variety of experimental conditions.

One limitation of the present review is that we did not compare the different techniques used to measure CBF. A number of different techniques were used in the studies mentioned in this review and some are known to be more accurate than others. However, the primary goal of the review was to take the techniques as a whole to determine what the consensus was on the effect of BS and CBF, and despite the variety of methods used, there was agreement amongst most of the studies. In future work, it may be beneficial to examine the different techniques used and their accuracy for measuring CBF.

Another limitation was that we focused on the quantitative effect that BS has on CBF but did not look at as much on the clinical implications of this in animals, in healthy controls or in patients with brain injury. For example, if certain anaesthetics are superior at reducing CMR_O2_, they might be more neuroprotective or if others have a high effect on CBF, they may not be the optimal choice for patients at risk for brain ischemia. In future work, we hope to look at the clinical significance of different anaesthetics and how this relates to their specific action on CBF.

Finally, in the present review, we were not able to conduct a meta-analysis of the data due to extremely heterogenous populations and data sets. In future work, it would be pertinent to narrow our search to studies in which there are more homogenous populations (e.g., patients with status epilepticus) and determine the effect size that BS has on CBF or even on clinical outcomes.

Future work should use *in vivo* methods of monitoring CBF such as NIRS in neurologically ill patients to determine the effect of BS in this population. The NIRS technique uses the infrared light, which can penetrate into deep tissues, and be absorbed by oxygenated and deoxygenated hemoglobin (Chen et al., 2020). The technique can determine the regional concentration of oxyhemoglobin and deoxyhemoglobin and thereby, the oxygenation of the tissue. In other words, NIRS is a cost-effective, portable, and efficient means of measuring neurovascular coupling. The advantage of being able to monitor cerebral physiology continuously and non-invasively in critically ill patients make it a favored technique for further investigating the questions left unanswered by the present review. A previous scoping review of 47 studies by our team showed evidence that continuous-wave NIRS metrics were linearly correlated with changes in CBF (Gomez et al., 2022). Cerebral oxygenation can be obtained from NIRS, and by correlating this value to MAP, one can obtain the cerebral oximetry index (COx) which is a measure of CA (Chen et al., 2020). When COx is close to zero or negative, autoregulation is being maintained but when the value is closer to one, autoregulation is impaired. Using NIRS, EEG, and BS, the effect of the above-discussed anaesthetics could be investigated in patients admitted to the neurological ICU in real time. Other evidence has shown that NIRS can predict epileptic spikes even before the spikes are seen on EEG (Chen et al., 2020). In patients with status epilepticus, the technique could be used to investigate CBF, autoregulation, and metabolism in this critically ill group of patients to determine how best to use BS to improve morbidity and mortality. Concurrent work by our group aims to answer these questions using continuous *in-vivo* methods of neuromonitoring such as NIRS.

### 4.4 Conclusion

In this systematic review of the effect of BS on CBF and autoregulation in animals and humans, we identified 67 studies. Most of the literature involved animal models, which largely involved small animals. In the human studies, most were conducted on patients undergoing surgery or who were critically ill, as expected. There was significant evidence to suggest that propofol and barbiturates decrease CBF in both animals and humans. However, the evidence regarding inhalational anaesthetics, comparative effects, and clinical implications is lacking. More large animal models as well as clinically relevant human studies are needed to further elucidate the mechanisms behind BS and CBF and what implications this has on the future of care in the neurological intensive care unit.

## Data Availability

The original contributions presented in the study are included in the article/Supplementary Material, further inquiries can be directed to the corresponding author.
