# Supplementary material for: The effect of burst suppression on cerebral blood flow and autoregulation: a scoping review of the human and animal literature
Source: Front Physiol. 2023 Jun 7;14:1204874. doi: 10.3389/fphys.2023.1204874 (PMC10282505; doi:10.3389/fphys.2023.1204874)
Supplement: Supplementary file 1 [file Table1.docx]

Supplementary Material

**The Effect of Burst Suppression on Cerebral Blood Flow and Autoregulation: A Scoping Review of the Human and Animal Literature**

A. Zohaib Siddiqi^1^, Logan Froese^2^, Alwyn Gomez,^3,^ Amanjyot Singh Sainbhi,^2^ Kevin Stein,^2^ Kangyun Park,^4^ Nuray Vakitbilir,^2^ Frederick A. Zeiler^2,3,5-7^

1. Department of Medicine, Rady Faculty of Health Sciences, University of Manitoba, Winnipeg, Canada
2. Biomedical Engineering, Faculty of Engineering, University of Manitoba, Winnipeg, Canada
3. Department of Human Anatomy and Cell Science, Rady Faculty of Health Sciences, University of Manitoba, Winnipeg, Canada
4. Undergraduate Medicine, Rady Faculty of Health Sciences, University of Manitoba, Winnipeg, Canada
5. Section of Neurosurgery, Department of Surgery, Rady Faculty of Health Sciences, University of Manitoba, Winnipeg, Canada
6. Department of Clinical Neuroscience, Karolinska Institute, Stockholm, Sweden
7. Division of Anaesthesia, Department of Medicine, Addenbrooke's Hospital, University of Cambridge, Cambridge, UK

*** Correspondence:**A. Zohaib Siddiqi, BSc MSc MD

Department of Internal Medicine, Rady Faculty of Health Sciences

University of Manitoba

Winnipeg, MB, Canada

Email: [siddiqia@myumanitoba.ca](mailto:Frederick.Zeiler@umanitoba.ca)

OrcID: <https://orcid.org/0000-0002-5228-6376>

# Supplementary Figures

Appendix A: Methods of Cerebral Autoregulation Included

| **Abbreviation** | **CA Method Description** |
| --- | --- |
| ARI | Autoregulatory Index – CA assessed by transfer function analysis of spontaneous fluctuations in ABP and FV |
| CBFx | Cerebral Blood Flow Index – correlation between CBF and CPP |
| CBFx-a | Cerebral Blood Flow Index – correlation between CBF and ABP |
| COx | Cerebral Oximetry Index – correlation between rSO_2_ and CPP |
| COx-a | Cerebral Oximetry Index – correlation between rSO_2_ and ABP |
| CTP | Computer Tomographic Perfusion – relies on central volume principle that relates CBF, CBV, and MTT |
| DCS | Diffuse Correlation Spectroscopy – CA assessed using direct measure of CBF using near-infrared light and metabolic rate can be assessed by pairing DCS with any NIRS system |
| DWI | Diffusion Weighted Imaging – CA assessed by using differences in Brownian motion to generate contrast using specific MRI sequences in correlation with TCDT |
| Dx | Diastolic Flow Index – correlation between FVd and CPP |
| Dx-a | Diastolic Flow Index – correlation between FVd and ABP |
| fMRI | Functional Magnetic Resonance Imaging – CA assessed by variations in regional tissue oxygenation using BOLD contrast agent with T2* weighted MRI |
| HVx/HBx | Hemoglobin Volume Index – correlation between relative total hemoglobin and ABP |
| LDx | Laser-Doppler Index – correlation between laser-Doppler flux and ABP |
| Lx | Correlation between LDF-based CBF and CPP |
| Lx-a | Correlation between LDF-based CBF and ABP |
| Mx | Mean Flow Index – correlation between FVm and CPP |
| Mx-a | Mean Flow Index – correlation between FVm and ABP |
| OHT | Orthostatic Hypotension Test – CA assessed by rapid change in head position while evaluating sudden drop in CBFV |
| PAx | Pulse Amplitude Index – correlation between AMP and ABP |
| PET | Positron Emission Tomography – CA assessed by evaluating positron emission decay using detector pairs from radiotracers |
| PRx | Pressure Reactivity Index – correlation between ICP and ABP |
| PWI-ASL | Perfusion Weighted Imaging - Arterial Spin Labelling – CA assessed by magnetically labeled blood for CBF measurements |
| PWI-DSC | Perfusion Weighted Imaging - Dynamic Susceptibility Contrast – CA assessed by administration of bolus of gadolinium-based contrast agent intravenously monitored using T2/T2*-weighted images |
| Sx | Systolic Flow Index – correlation between FVs and CPP |
| Sx-a | Systolic Flow Index – correlation between FVs and ABP |
| THRT | Transient Hyperemic Response Test – CA assessed by compression of carotid artery while insonating ipsilateral MCA |
| THbx | Tissue Hemoglobin Index – correlation between |
| THx | Total Hemoglobin Index – correlation between THI and CPP |
| THx-a | Total Hemoglobin Index – correlation between THI and ABP |
| TOx | Tissue Oxygen Index – correlation between TOI and CPP |
| TOx-a | Tissue Oxygen Index – correlation between TOI and ABP |
| Xe-CT | Xenon-Computer Tomography – CA assessed by CT scans during inhalation of xenon gas mixture where Kety-Schmidt equations are used to calculate CBF |
| ABP, arterial blood pressure; AMP, pulse amplitude of ICP; BOLD, blood oxygen level dependent; CA, cerebral autoregulation; CBF, cerebral blood flow; CBFV, cerebral blood flow velocity; CBV, cerebral blood volume; CPP, cerebral perfusion pressure; CT, computed tomography; FV, flow velocity; FVd, diastolic flow velocity; FVm, mean flow velocity; FVs, systolic flow velocity; Hb, deoxyhemoglobin; HbO, oxyhemoglobin; ICP, intracranial pressure; LDF, laser-doppler flow; MAP, mean arterial pressure; MCA, middle cerebral artery; MRI, magnetic resonance imaging; MTT, mean transit time; NIRS, near-infrared spectroscopy; PbtO_2_, brain tissue oxygenation; rSO_2_, regional cerebral oximetry; TCDT, thigh cuff deflation technique; THI, total hemoglobin index; TOI, total oxygen index. | |

Appendix B: Search Strategy for BIOSIS

TITLE-ABS-KEY (( "Deep Sedation" OR "CNS depression" OR "Central nervous system depression" OR "Burst-suppression" OR "Suppression Burst" OR "BS" OR "EEG suppression" OR "Electroencephalogram" OR "Electrocerebral Silence" OR "Low-voltage EEG" OR "Low-voltage electroencephalogram" OR "Deep Metabolic Suppression" )

AND

("CBF" OR "Brain Blood Flow" OR "Cerebral Blood Flow" OR "Cerebral hemodynamics" OR "Cerebral haemodynamics" OR "Cerebral homeostasis" OR "Cerebral circulation" OR "Cerebral microcirculation" OR "Cortical blood flow" OR "Cerebral pressure autoregulation" OR "Cerebral Vasoreactivity" OR "Cerebral artery flow velocity" OR "Cerebrovascular control" OR "Brain Perfusion" OR "Cerebral Perfusion Pressure" OR "Cerebral perfusion" OR "Cerebral metabolism" OR "Cerebral pressure" OR "Cerebral vasculature" OR "Cerebral vasodilation" OR "Cerebral vasoconstriction" OR "Cerebral metabolism" OR "CA" OR "Cerebral Flow metabolism" OR "Neurovascular coupling" OR "Neurovascular autoregulation" OR "Neurovascular reactivity" OR "Blood Flow Velocities" OR "Blood Flow Velocity" OR "Cerebral vascular resistance" OR "Cerebral vascular reactivity" OR "Cerebral vasomotor responsiveness" OR "Cortical perfusion" OR "Transcranial Doppler" OR "TCD" OR "Cerebrovascular Function" OR "Pulsatility Index" OR "Brain Autoregulation" OR "Cerebrovascular reactivity" OR "Transcranial Doppler Sonography" OR "Pressure Reactivity index" OR "PRx" OR "Mean flow index" OR "Mx" OR "Pulsatile Reactivity index" OR "PAx" OR "Autoregulation index" OR "ARI" OR "Transfer function analysis" OR "TF" OR "TFA" OR "Cerebral Oximetry index" OR "COx" OR "Tissue Oxygenation index" OR "TOx" OR "Projection pursuit regression" OR "PPR" OR "Cerebral spinal reserve capacity" OR "RAP" OR "Spatially resolved NIRS" OR "Transcranial doppler index" OR "TCDx" OR "Flow velocity" OR "FV" OR "Systolic flow index" OR "Sx" OR "Diastolic flow index" OR "Dx" OR "hemoglobin volume index" OR "HBx" OR "tissue hemoglobin index" OR "tHbx" OR "Dynamic autoregulatory index" OR "DAx" OR "Laser Doppler flowmetry" OR "LDF" OR "brain tissue oxygen tension" OR "PbtO2" OR "Thermal Diffusion" OR "TDx" OR "Computed tomographic Perfusion" OR "CTp" OR "Mean transit time" OR "MTT" OR "Time to peak" OR "TTP" OR "Xe-CT" OR "Xenon-CT" OR "Spatially resolved NIRS" OR "Diffusion Weighted Imaging index" OR "DWI" OR "Perfusion-weighted imaging index" OR "Perfusion-weighted imaging" OR "dynamic susceptibility Contrast" OR "PWI DSC" OR "Perfusion-weighted imaging" OR "Arterial spin labeling" OR "PWI ASL" OR "Functional MRI" OR "fMRI" OR "Positron Emission Tomography" OR "PET" OR "Diffuse Correlation Spectroscopy" OR "DCS" OR "arteriovenous difference in oxygen" OR "AVDO2" OR "Thigh cuff deflation technique" OR "TCDT" OR "transient hyperemic response test" OR "THRT" OR "orthostatic hypotension test" OR "OHT" OR "oxygen extraction fraction" OR "OEF" OR "Animal CBF" OR "Cortical laser doppler" OR "Radiolabeled microspheres" OR "Kety--Schmidt technique" OR "Arterio--venous difference" OR "AVDO2" OR "arterio-jugular oxygen content" OR "AJDO2" OR "cerebral metabolic rate for oxygen" OR "CMRO2" OR "Jugular bulb saturation" OR "SjO2⁠" OR "radiolabeled microspheres" OR "radioactive inert gases" OR "Gas Clearance" OR "Cerebral Vessel Diameter")

AND NOT

("Non English" OR "Non-English" OR "Cadaver Studies" OR "Theoretical studies")

# Tables

**Supplementary Table 1:** **Summary of articles examining the effect of BS on CBF in animals.**

| **Article** | **Study Subjects** | **Experimental Conditions** | **Number of Subjects** | **Method of BS** | **BS Determination** | **Measure of CBF** | **Study Results and Conclusions** | **Study Limitations** |
| --- | --- | --- | --- | --- | --- | --- | --- | --- |
| Propofol |  | | | | | | | |
| Artru et al., 1992 (1) | Mongrel Dogs | In a group of dogs, CBF, CMR_O2_, CVR, and ICP were measured at different concentrations of propofol (12, 24, and 48 mg/kg/hr) and under normocapnia compared with hypocapnia, with and without addition of phenylephrine. | 12 | Propofol | EEG power spectrum analysis | Measuring blood flow in sagittal sinus  CMR_O2_= CBF X (O2_SA_-O2_SS_)  CVR= CPP/CBF  CPP= MAP-ICP | - BS EEG was obtained when propofol reached the highest concentration (48 mg/kg/h). At this dose, there was a significant decrease in MAP by 43%. - During normocapnia, there was a significant decrease in CBF in all propofol concentrations, the largest decrease being at the BS dose (76%, p<0.05). The same was true for CMR_O2,_ BS causing a 31% reduction (p<0.05). - With addition of phenylephrine, there was no significant difference in cerebral vascular resistance but the decrease in CBF was blunted (61%, p<0.05) but there was no blunting of the CMR_O2_ decrease. - During hypocapnia, there was a significant decrease in CBF in all propofol concentrations, the largest decrease being at the BS dose (77%, p<0.05). The same was true for CMR_O2,_ BS causing a 24% reduction (p<0.05) - With addition of phenylephrine, there was no significant difference in cerebral vascular resistance but the decrease in CBF was blunted (49%, p<0.05) but there was no blunting of the CMR_O2_ decrease. | - Regional CBF measurements not acquired - CVR not measured but calculated indirectly |
| Joshi et al., 2006 (2) | New Zealand White Rabbits | In a group of rabbits, the dose of propofol to induce isoelectric EEG and CBF was measured under 3 conditions: changes in ventilation, addition of verapamil, and changes in cerebral perfusion. | 32 | Intracarotid Propofol (1%) | EEG Visualized | Laser doppler flowmetry | - In the second two conditions, the results pre verapamil and pre hypoperfusion are reported. - In the first group, when the EEG was made isoelectric, MAP significantly decreased by 9%. CBF nonsignificantly decreased by 8% in the ipsilateral hemisphere and significantly by 18% in the contralateral hemisphere (p<0.0167). During recovery of EEG and BS, MAP did not significantly change. CBF significantly decreased by 19% in the ipsilateral hemisphere and 22% in the contralateral hemisphere (p<0.0167) - In the second group, with isoelectric EEG, MAP was 16% lower than baseline. CBF significantly decreased by 16% in the ipsilateral hemisphere and 19% in the contralateral hemisphere (p<0.0167). With recovery (BS), there was no significant difference in MAP compared to baseline. CBF was 19% significantly lower than baseline in the ipsilateral hemisphere (p<0.0167) but there was no significant change in the contralateral hemisphere - In the third group, there was no significant change in CBF in either hemisphere with isoelectric EEG or recovery | - No measurement of CMR_O2_ limiting conclusions that can be drawn - BS measured by visualization limiting accuracy |
| Liu et al., 2013 (3) | Sprague Dawley Rats | In a group of rats, BS was induced, and functional connectivity of brain regions of interest assessed by fMRI. | 7 | IV Propofol (20-100mg/kg/hr) | EEG Power spectral analysis and BS ratio | fMRI BOLD signal/functional connectivity analysis | - BS ratio increased as propofol dose increased (0.1 with 20mg/kg/hr to 0.7 at 100mg/kg/hr) with most animals reaching BS at doses of 80 and 100mg/kg per hour - There was no significant difference in MAP with propofol infusion - At the 20mg/kg/hr dose, there was widespread functional connectivity with a relatively greater volume compared to other doses. There was fluctuation of functional connectivity as the propofol dose was increased (p<0.05). - Subcortical connectivity increased with BS (p<0.05) | - Functional connectivity analysis measures CBF indirectly limiting accuracy - Conclusions that can be drawn about CBF from functional connectivity analysis are limited |
| Ramani et al., 1992 (4) | New Zealand White Rabbits | In a group of rabbits, isoelectric EEG was induced by propofol and CBF and CMR_O2_ were measured at different concentrations and time points. | 8 | Propofol | EEG Power Analysis | Hydrogen Clearance Method  CMR_O2_= CBF X (O2_SA_-O2_SS_) | - MAP decreased 10% despite the use of angiotensin II to maintain MAP (p<0.05) - Isoelectric EEG occurred in only 2 rabbits at 41 and 52 μg/mL - As propofol blood level increased, CBF and CMR_O2_ fell, reaching 62% and 57% of baseline respectively, at a blood level 40 μg/mL (p value not stated) - When plotted against EEG power, CMR_O2_ was 53% when EEG was isoelectric | - Very low sample size of animals that reached isoelectricity - CBF measured indirectly, limiting accuracy - No analysis of relationship between EEG power and CBF - No significance stated for drop in CBF and CMR_O2_ |
| Wang et al., 2011 (5) | New Zealand White Rabbits | In a group of rabbits, EEG was made isoelectric and CBF in the ipsilateral and contralateral hemisphere was measured during intracarotid and intravenous propofol injection in an ischemia-reperfusion model. | 11 | Intracarotid/Intravenous Propofol 1%  200–400 μg/k1g/min | EEG Visualized | Laser Doppler Flowmetry  NADH (marker of O2 metabolism) | - There was no significant difference in MAP between control, intracarotid, and intravenous propofol in any of the conditions - Hypoperfusion led to a significant decrease in CBF in ipsilateral and contralateral hemisphere (p<0.0167) but there was no significant difference between control and propofol groups. - Five and ten minutes after hypoperfusion, there was a significant hyperemic response (p<0.0167). There was no difference between control, intracarotid, or intravenous propofol groups in the amount of CBF recovery. - There was no significant difference in NADH levels in either of the 3 groups in any of the 3 conditions | - No comparison of CBF at baseline versus IV/intracarotid propofol, limiting conclusions that can be drawn - Isoelectric EEG measured by visualization, limiting accuracy - Cerebral metabolism measured indirectly, limiting accuracy |
| Werner et al., 1992 (6) | Mongrel Dogs | In a group of dogs, BS was induced by propofol and regional CBF, mean CBF velocity, ICP, PI, CVR, and CMRO2 were measured. | 11 | Propofol | EEG visualized | Quantitative autoradiography for CBF  TCD for MCA CBF velocity  PI=V_systolic_-V_diastole_  CVR = (MAP-ICP)/CBF  CMR_O2_= CBF X (O2_SA_-O2_SS_) | - There was no significant change in MAP with propofol - When BS was achieved, CBF in cortex and caudate as well as CMR_O2_ decreased by 70% (p<0.05). - When propofol was decreased and EEG activity returned, CBF and CMR_O2_ increased to 55% of baseline (p<0.05) and subsequently returned to 30% of baseline when BS was induced again (p<0.05) - When BS was achieved, CVR increased by 210% and PI increased by 50% (p<0.05) | - BS measured by visualization limiting accuracy - CBF measured indirectly, limiting accuracy |
| Barbiturates |  |  |  |  |  |  |  |  |
| Gronert et al., 1981 (7) | Mongrel Dogs | In a group of 16 mongrel dogs, BS was induced and CBF measured at different time points in different dogs. | 16 | Pentobarbital | EEG visualized | Measuring blood flow in sagittal sinus  CMR_O2_= CBF X (O2_SA_-O2_SS_) | - MAP dropped at sequential time points, at its lowest point being 56% lower than the highest value - After the baseline 0-3h condition, there was an abrupt 25% increase (p<0.05) in CMR_O2_ which stayed consistent in all subsequent time periods (3-6h, 12-15h, and 21-24h) - CBF decreased progressively across all time points | - BS measured by visualization limiting accuracy - Average value of CBF not calculated, making it challenging to interpret how much CBF decreased after baseline measurements - Different dogs used in the 0-3h condition vs other conditions, limiting conclusions that can be drawn |
| Hungerhuber et al., 2006 (8) | Sprague-Dawley Rats | In a group of rats, BS was induced by the barbiturate methohexital and CBF was measured at baseline and in an ischemia-reperfusion model. | 35 | IV Methohexital (1.0–1.5 mg/kg/min) | EEG Visualized | Laser doppler flowmetry | - In the BS group, there was an 80% significant decrease in CBF in the ipsilateral region by 80% and 70% in the contralateral hemisphere (p<0.05) - Occlusion of the MCA resulted in significantly 20% significant reduction in CBF in the ipsilateral MCA territory without any change in the contralateral territory. There was no significant difference in CBF during reperfusion in rats treated burst-suppressed rats compared to non-burst-suppressed rats | - MAP not measured so unclear if drop in CBF was also associated with drop in MAP - CMR_O2_ not examined - BS measured by visualization, limiting accuracy |
| Joshi et al., 2005 (9) | New Zealand White Rabbits | In a group of 9 rabbits, BS was induced and the resulting CBF in the ipsilateral and contralateral hemisphere was measured with and without cerebral hypoperfusion | 9 | Intracarotid Thiopental (1%) | EEG Visualized | Laser doppler flowmetry | - There was no significant decrease in MAP with thiopental injection alone. During hypoperfusion, MAP decreased by 67% (p<0.0001) - During hypoperfusion, there was a 69% decrease in ipsilateral CBF and 53% decrease in contralateral CBF (p<0.005) - There was no significant change in CBF with injection of thiopental alone. | - BS measured by visualization limiting accuracy - No measurement of CMR_O2_ limiting conclusions that can be drawn |
| Kassell et al., 1980 (10) | Mongrel dogs | In a group of dogs, BS was induce and CBF, CMR_O2_, and CVR were measured at time points of 30, 60, 120, and 240 seconds. | 9 | Sodium thiopental | EEG visualized | Quantitative autoradiography  CMR_O2_= CBF X (O2_SA_-O2_SS_)  CVR= MAP-SSP/CBF | - MAP decreased by approximately 32% (no p value given) - With induction of BS (from 0-30 s), total CBF decreased by 45% and CMR_O2_ by 42%. CVR increased by 125%. - CBF, CMR_O2_, and CVR reached a plateau at BS between 30 and 60 seconds. - There was a strong positive correlation between CBF and CMR_O2_ (R= 0.77; p<0.05). - There was a strong negative correlation between CVR and CMR_O2_ (R= -0.77; p<0.05). | - BS measured by visualization, limiting accuracy - CBF measured indirectly, limiting accuracy |
| Klementavicius et al., 1996 (11) | Wistar Rats | In a group of rats, isoelectric EEG was attained and CBF was measured at normothermia (38°C) and hypothermia (34°C, 20°C, and 28°C). | 11 | Thiopental Sodium | EEG Visualized | Hydrogen Clearance method  CMR_O2_= CBF X (O2_SA_-O2_SS_) | - MAP was kept above 100mmHg with donor blood but did fall 14% with thiopental during normothermia and 25% during hypothermia (no significance reported) - In the normothermic rats (n=5), both CBF and CMR_O2_ decreased by 50% after isoelectric EEG was attained (p<0.05) - In the hypothermia groups (n=6), CMR_O2_ decreased by 46% in the 34°C condition, 74% in the 30°C condition, and 80% in the 28°C condition when isoelectricity was attained (p<0.05) - After attainment of isoelectric EEG, the ratio of CBF:CMR_O2_ increased from 20.9 to 32.1 as temperature fell from 38°C to 28°C. This indicated that the reduction in CMR_O2_ was greater than that in CBF (p<0.05) | - CBF measured indirectly, limiting accuracy - BS measured by visualization limiting accuracy |
| Makiranta et al., 2002 (12) | Piglets | In a group of piglets, BS was induced and CBF and cerebral activity were measured by fMRI in the whole brain, cortex, and thalamus regions of interest (ROIs). | 5 | Thiopental (11.4-17.1 mg/kg) | EEG Power Analysis | fMRI BOLD signal analysis | - EEG showed burst-suppression state 4 min after thiopental injection - MAP decreased by 25% - There was an increase in total averaged signal variance after the thiopental injection in the whole brain ROI. The cortical ROI showed no change. There was a positive spike of 0.5% signal change in the thalamus. - The group averages of the total ROI responding voxels showed that in the thalamus, the negative response (-3% to -4%). was less clear than the positive (6% to 8%). In the whole brain and cortex ROIs, both negative (-6% to -8%) and positive responses (6% to 8%). were seen. | - Low sample size - No p values reported - Challenging to interpret the results in the context of CBF and CMR_O2_, limiting conclusions that can be drawn - Challenging to isolate the specific effect of BS on FMRI signal or CBF, limiting conclusions that can be drawn |
| Michenfelder, 1974 (13) | Mongrel Dogs | In a group of dogs, isoelectric EEG was induced and CBF, CVR CMR_O2_, and CMR_glu,,_ at different time points and levels of EEG suppression. | 7 | Thiopental (177mg/kg) | EEG Visualized | Measuring blood flow in sagittal sinus  CMR_O2_= CBF X (O2_SA_-O2_SS_)  CMR_glu_= CBF x (Glu_art_- Glu_v_)  CVR=MAP/CBF | - Isoelectric EEG was attained at 72mg/kg - Despite infusion of donor blood, MAP reduced significantly by 40% during infusion (p<0.001) - At isoelectric EEG, CBF decreased by 55% (p<0.001) and CMR_O2_ by 42% (p<0.001). There was no further change in CMR_O2_ after isoelectric EEG was reached. - With the assumption that the initial halothane given had already decreased CMR_O2_, the adjusted value of CMR_O2_ decrease was 54% - CVR increased by 34% (p<0.02), and CMR_glu_ decreased by 45% (p<0.01) | - Small sample size - BS measured by visualization, limiting accuracy |
| Milde et al., 1985 (14) | Dogs | In a group of dogs, BS was induced and CBF, CMR_O2_, CVR, and ICP measured at different time points and concentrations of Etomidate. | 6 | Etomidate (0.02-0.4 mg/kg/min) | EEG Visualized | Measuring blood flow in sagittal sinus  CMR_O2_= CBF X (O2_SA_-O2_SS_)  CVR= MAP/CBF  CPP=MAP-ICP | - BS and isoelectric EEG were attained at 0.2 and 0.3 mg/kg/min respectively - MAP decreased during infusion, reaching a trough of 29% of baseline at the end (p<0.05) - CMR_O2_ decreased by 54% (p<0.05), when isoelectric EEG was obtained and thereafter plateaued - CBF decreased by 75% (p<0.05) and plateaued before BS was attainted. - CVR was inversely related to CBF, increasing to 214% of baseline when CBF was at 50% of baseline, and increasing to 300% of baseline when CBF reached its trough (p<0.05). The decrease in increase in CVR was also accompanied by a decrease in ICP. | - Low sample size - BS measured by visualization, limiting accuracy - Difficult to compare changes in MAP to changes in CBF by the way results are reported in the study |
| Nemoto et al., 1996 (15) | Wistar Rats | In two groups of rats, isoelectricity was induced by thiopental and CBF and CMR_O2_ were measured at normothermia and hypothermia. | 14 | Thiopental 15-20mg | EEG visualized | Hydrogen Clearance Method  CMR_O2_= CBF X (O2_SA_-O2_SS_) | - Thiopental was titrated to induce isoelectricity in both groups of rats - MAP was 26% significantly lower with thiopental infusion during normothermia (p<0.01) and 36% lower with thiopental infusion during hypothermia (p<0.01) - In the normothermic condition, when EEG was isoelectric, CBF decreased by 49% and CMR_O2_ by 50% (p<0.05) - In the hypothermic condition, when EEG was isoelectric, CBF was reduced by 56% and CMR_O2_ by 64% (p<0.05) | - BS was measured using visualization, limiting accuracy - CBF was measured indirectly, limiting accuracy - MAP decreased in thiopental group, limiting conclusions that can be drawn |
| Nemoto et al., 1996 (16) | Wistar Rats | In two groups of rats, isoelectricity was induced by thiopental and MAP was maintained by norepinephrine or donor blood; measurements were made at normothermia and mild hypothermia. | 12 | Thiopental 15-20mg | EEG visualized | Hydrogen Clearance Method  CMR_O2_= CBF X (O2_SA_-O2_SS_) | - When donor blood was used to support MAP, there was a 26% drop and when norepinephrine was used there was a 15% drop (p<0.05) - In normothermic rats, there was 50% reduction in CBF when donor blood was used and a 65% reduction in CBF when norepinephrine was used (p<0.05) - In normothermic rats, there was 50% reduction in CMR_O2_ when donor blood was used to support MAP and a 48% reduction when norepinephrine was used (p<0.05) - In hypothermic rats, there was 61% reduction in CBF when donor blood was used to support MAP and a 44% reduction in CBF when norepinephrine was used (p<0.05) - In hypothermic rats, there was 62% reduction in CMR_O2_ when donor blood was used to support MAP and a 29% reduction in CMR_O2_ when norepinephrine was used (p<0.05) | - BS was measured using visualization, limiting accuracy - CBF was measured indirectly, limiting accuracy |
| Schmid-Elsaesser et al., 1999 (17) | Sprague-Dawley Rats | In a group of rats, BS was induced by thiopental, and CBF and infarct volume was compared between control (halothane), non-BS isoflurane, and burst-suppression isoflurane groups in an ischemia-reperfusion model. | 30 | Thiopental 26–40 mg/kg/h | EEG Visualized | Laser Doppler flowmetry  Measurement of infarct volume | - Non-BS doses of isoflurane significantly reduced CBF by 20% (p<0.05). BS doses of thiopental significantly reduced CBF by 30% (p<0.05) - Compared with control (halothane) groups, isoflurane groups had 28% significantly less infarct volume (p<0.01). There was no significant difference in infarct volume between non-burst-suppression isoflurane or burst-suppression isoflurane groups | - CMR_O2_ not measured - BS was measured by visualization, limiting accuracy - MAP not measured |
| Westermaier et al., 2000 (18) | Sprague Dawley Rats | In a group of rats, CBF was measured before and after induction of BS, during normothermia and hypothermia, and with and without MCA occlusion. | 32 | IV Methohexital (1-1.5 mg/kg/min) | EEG Visualized | Laser Doppler Flowmetry | - There was no significant difference in MAP across groups - In normothermic rats without BS, MCA occlusion resulted in a significant 70-80% reduction in CBF in the ipsilateral hemisphere (p<0.05) and no reduction in the contralateral hemisphere. - In the normothermic condition, BS resulted in a 30% decrease in CBF in the ipsi- and contra-lateral hemisphere (p<0.05). Occlusion of the MCA caused ipsilateral CBF to drop by 80% without any change in the contralateral hemisphere - In hypothermic rats without BS, ipsi- and contra-lateral CBF decreased by 20% (p<0.05). MCA occlusion resulted in a 70-80% reduction in CBF in the ipsilateral hemisphere and no reduction in the contralateral hemisphere (p<0.05). - In hypothermic rats with BS, CBF decreased by 50% (p<0.05) in the ipsi- and contra-lateral hemisphere. With MCA occlusion, ipsilateral CBF decreased by 80% without any effect on the contralateral hemisphere (p<0.05). - BS alone did not have any significant effect on infarct volumes | - BS measured by visualization limiting accuracy - No measurement of CMR_O2_ |
| Zarchin et al., 1998 (19) | Mongolian Gerbils | In a group of gerbils, BS was induced and CBF and NADH levels were measured before and after cerebral ischemia was induced. | 14 | Thiopental 7.5mg | (intracranial) EEG Visualized | Laser Doppler Flowmetry  NADH levels (marker of oxygen metabolism) | - After induction of BS, CBF was reduced by 23% compared to control (p<0.05) - After ischemia was induced, there was no significant difference in the reduction of CBF in the BS group (84.3%) to the control group (88.5%) - The increase in NADH levels following ischemia was significantly higher in the control group compared to the BS group (90% vs. 65.75%, p<0.05). | - BS was measured by visualization, limiting accuracy - MAP not measured, limiting conclusions that can be drawn |
| Inhalational |  |  |  |  |  |  |  |  |
| Baughman et al., 1989 (20) | Sprague Dawley Rats | In a group of rats, the effect of isoflurane and nitric oxide was tested on CBF and CMR_O2_ at different severities of brain ischemia_._ In a separate group of rats, the effect of the anaesthetics and ischemia was examined on EEG. | 50/8 | Isoflurane, nitric oxide, ischemia | EEG visualized | Quantitative autoradiography  CMR_O2_ measured by sampling sagittal sinus blood | - With isoflurane alone or with isoflurane plus ischemia, there was never BS or isoelectric EEG - At 0.5 MAC isoflurane CBF was 44% decreased and CMR_O2_ was 33% reduced (p<0.05) and at 1 MAC isoflurane, CBF was reduced by 47% and CMR_O2_ by 17% (p<0.05) - The reduction of CBF caused by 0.5 MAC isoflurane was completely reversed with addition of 70% nitric oxide. - The reduction of CBF caused by 1.0 MAC was partially reversed with addition of nitric oxide | - BS never attained - EEG and CBF measurements performed on separate rats, limiting conclusions that can be drawn - CBF measured indirectly, limiting accuracy |
| Benveniste et al., 2017 (21) | Fisher and Sprague Dawley Rats | In a group of rats, measurements of glymphatic transport were made in rats anaesthetized with isoflurane alone compared with rats anaesthetized with isoflurane + dexmedetomidine (ISOdex). | 32 | Isoflurane (2-3%) | EEG Visualized | Analysis of glymphatic transport and contrast uptake  MR Time of Flight for vessel diameters | - BS was seen only in rats anesthetized with isoflurane and not in the ISOdex rats - Isoflurane produced greater vasodilation in the straight sinus compared to the iso ISOdex rats but not in the superior sagittal sinus, external jugular vein, or internal carotid artery (p=0.002) - Isoflurane rats had 32% less uptake of contrast agent gadopentetic acid than ISOdex rats (p<0.003) - The peak magnitude of contrast in the isoflurane group was 59% less than in the ISOdex group(p=0.0017). The calculated clearance rate was 75% less in the isoflurane group compared with the ISOdex group (p=0.0011). - The volume of CSF in the isoflurane group was 36% significantly less than the volume of CSF in the ISOdex group (p<0.01) | - EEG measured in separate rats compared to those in which other parameters were measured, limiting accuracy and conclusions that can be drawn - Contrast and lympathic measurement indirect measurement of CBF, limiting accuracy - BS measured by visualization limiting accuracy - MAP values not reported, limiting conclusions that can be drawn |
| Berndt et al., 2021 (22) | Wistar Rats | In a group of rats, BS was induced and cortical tissue oxygen pressure (CTOP) and CBF was measured. | 13 | Isoflurane (1-2%) | (intracranial) EEG visualized | Laser doppler flowmetry  O_2_ electrode for CTOP measurements | - There was no significant change in MAP with increasing depth of anaesthesia - There was a 35% decrease in CBF in the BS state than when the EEG showed delta/alpha activity. During BS, CBF bursts were closed coupled with EEG bursts (p<0.001) - There was 45% more CTOP in the BS state than when the EEG showed delta/alpha activity (p=0.02). Every EEG burst, there was a 5mmHg spike in cortical tissue oxygen pressure. | - Comparisons in CTOP and CBF not performed versus baseline condition |
| Choi et al., 2016 (23) | Long Evans Rats | In a group of rats, BS was induced at different concentrations of isoflurane and resulting changes in CBF measured. | 8 | Isoflurane (1.5%-2.5%) | Local Field Potentials and BS Ratio | Near Infrared Spectroscopy  Change in totalHgb= oxyHgb+ change in deoxyHgb | - As the concentration of isoflurane increased, the duration of BS, total Hgb (R^2^=0.53 ,p<0.05), and oxyHgb significantly increased (R^2^=0.72, p<0.05), and the amount of deoxyHgb significantly decreased (R^2^=-0.92; p<0.05). | - Low sample size - CBF measured indirectly, limiting accuracy - BS measured indirectly, limiting accuracy |
| Golanov and Reis, 1995 (24) | Sprague-Dawley Rats | In a group of rats, BS was induced and regional CBF was measured in cerebellar fastigial nuclear and ventrolateral medulla. | 41 | Isoflurane 1.5% to 1.7% | (intracranial) EEG Visualized | Laser Doppler Flowmetry | - During BS, there was an increase in regional CBF of 20% compared to baseline during the “bursts” (p<0.05). This increase gradually recovered over 10s. - The latency between the onset of a “burst” and the onset of the increase in regional CBF was 1.2s, the peak was reached at 4.5s, and the entire event lasted 12s, that is to say, there were 5-6 events per minute. - Injection of procaine into subdural space, decreased amplitude of electrical bursts but maintained BS. - Injection of procaine eliminated the spontaneous rises in CBF that occurred with spikes during BS | - CBF not measured before BS was induced, limiting the conclusions that can be drawn - Cerebral metabolism not measured or calculated - BS measured by visualization, limiting accuracy |
| Kochs et al., 1993 (25) | Mongrel Dogs | In a group of dogs, BS was induced and ICP, CBF, CBF velocity, and CMR_O2_ were measured at different concentrations and times. | 13 | Isoflurane | EEG Power Analysis | Quantitative Autoradiography  TCD for CBF Velocity  CMR_O2_= CBF X (O2_SA_-O2_SS_) | - Burst-suppression patterns was attained when isoflurane concentration was >2% and isoelectricity was attained at 3% concentration. - MAP was maintained with phenylephrine and there was no significant change - There was a significant 35% increase in ICP with 2% isoflurane (p<0.05) and significant 65% increase in ICP at 3% isoflurane (p<0.05) - At 1% isoflurane concentration, there was no significant change in CBF. At 2%, the CBF significantly increased by 36% (p<0.05) and at 3% it increased by 106% (p<0.05). - Compared to control, there was no significant change in CBF velocity at isoflurane 1% concentration but there was a significant 20% increase at 2% (p<0.05) and significant 71% increase at 3% ( p<0.05) - The correlation between relative changes in CBF velocity and CBF over all isoflurane concentrations was r = 0.94 (P < 0.001). - A negative correlation was found between periods of complete EEG suppression and CMR_O2_ (r = -0.73, p< 0.05). | - Addition of nitric oxide has been shown to neutralize effect that isoflurane has on CBF, limiting the conclusions of the data - CBF was measured indirectly, limiting accuracy |
| Liu et al., 2011 (26) | Sprague-Dawley Rats | In a group of 6 rats, burst suppressed was induced and CBF measured by laser doppler flowmetry at three concentrations. The experiments were repeated in 10 rats using fMRI. | 6/10 | Isoflurane (1.8%, 2%, 2.2%) | EEG Visualized | Laser Doppler Flowmetry  fMRI BOLD signal | - BS was attained at 1.8% concentration. As concentration was increased, appearance of spikes was reduced and at 2.2%, only rare single spikes were seen. - Compared to the 1.8% condition, there was a 5.3% decrease in MAP in the 2.2% condition (unclear if significant) - In the LDF experiments, there was significant correlation between spikes of CBF increases and EEG spikes in the 1.6% and 2% concentration conditions (r=0.61 ± 0.17 and r= 0.65 ± 0.20 respectively; p<0.01) - Correlation was significantly reduced in the 2.2% concentration condition (r= to 0.28 ± 0.21) and was absent when EEG was silent after animal sacrifice (r=0.02 ± 0.07, not significantly different from 0) - In the fMRI experiments, in the 1.8% and 2.0% conditions, there was strong correlation between EEG spikes and BOLD signal spikes in several brain regions relevant in sensorimotor functions. The correlation was much weaker in the 2.2% condition. - There was strong linear correlation between LDF and BOLD responses (R^2^ =0.96) and between EEG-predicted LDF and BOLD signals R^2^=0.99 (p<0.05) - When the baseline LDF and BOLD signal were normalized by those of the 1.8% condition, there was no significant difference in signal between the concentration groups | - No description of signal before and after induction of BS, limiting conclusions that can be drawn - BS measured by visualization limiting accuracy |
| Lutz et al., 1990 (27) | Beagle dogs | In a group of dogs, BS was induced by desflurane and CBF, CMR_O2_, and ICP were measured at different concentrations, with and without addition of phenylephrine. | 6 | Desflurane | EEG visualized | Measuring blood flow in sagittal sinus  CMR_O2_= CBF X (O2_SA_-O2_SS_) | - BS was attained at 2.0 MAC desflurane but this EEG activity returned with time - Desflurane produced a 56% drop in MAP. With addition of phenylephrine, the drop was 32% (p<0.05) - Without phenylephrine, CBF increased at 0.5, 1.0, and 1.5 MAC. At 2.0 MAC, there was a 29% drop in CBF (p<0.05). With addition of phenylephrine, there was no significant different in CBF from 1.5 MAC to 2.0 MAC - With increasing concentrations of desflurane, there was a decrease in CMR_O2,_ reaching a trough at 29% below that of the 0.5 MAC level). This was not reversed with addition of phenylephrine - ICP was lowest in the 2.0 MAC condition (53% less than that of 0.5 MAC level). Phenylephrine had no significant effect on ICP. | - No measure of CBF at baseline, without desflurane - BS disappeared over time, limiting conclusions that can be drawn - Only significance values of conditions with phenylephrine calculated - BS measured by visualization limiting accuracy |
| Maekawa et al., 1986 (28) | Sprague-Dawley Rats | In a group of 61 rats, BS was induced and CBF and lCMR_glu_ was measured at different concentrations of isoflurane in different groups of rats. | 61 | Isoflurane | EEG visualized | Quantitative autoradiography | - BS occurred at concentrations of 1.0 and 1.5 MAC and isoelectricity occurred at 2.0 MAC - MAP significantly decreased at 1.0 MAC (26%, p<0.01), 1.5 MAC (29%, p<0.001), and 2.0 MAC (36%, p<0.001) compared to baseline - There was no significant difference in CBF between controls and 1.0 MAC condition - At 1.5 MAC, the auditory cortex saw a 4.9% significant decrease in CBF (p<0.05), the visual cortex did not see any significant change, and the extrapyramidal system (140-170%) and limbic system (87%-120%) saw a significant increase (p<0.01) - In the 2.0 MAC condition, all regions saw a significant increase in CBF (highest in superior colliculus of visual system, 225%, p<0.01) - There were significant decreases in lCMR_glu_ at all regions except hippocampus and corpus callosum in 1.0, 1.5, and 2.0 MAC conditions (p<0.01, p<0.05) | - Different concentrations were investigated in different groups of rats, limiting conclusions that can be drawn - Small sample sizes in each individual concentration group - BS measured by visualization limiting accuracy - CBF measured indirectly, limiting accuracy |
| Newberg et al., 1983 (29) | Mongrel Dogs | In a group of 9 dogs, BS was induced by different concentrations of isoflurane and CBF and CMR_O2_, and CVR were measured at these concentrations. | 9 | Isoflurane (3%) | EEG visualized | Measuring blood flow in sagittal sinus  CMR_O2_= CBF X (O2_SA_-O2_SS_)  CVR= MAP/CBF | - At 1.4% concentration, there was slowing but no BS. At 3.0% BS was seen. At 6.0% the interval between bursts and isoelectricity widened. - MAP significantly decreased by 24% in the 3.0% condition and by 31% in the 6.0% condition (p<0.05) - There was no significant change in CBF in all three conditions. - There was a significant 28% decrease in CMR_O2_ when isoflurane was increased from 1.4% to 3.0% (p<0.05) but no significant change from 3.0% to 6.0%. - There was no significant change in CVR from the 1.4% condition to 3.0% but there was a significant 41% decrease from the 1.4% condition to the 6.0% condition (p<0.05) - Increasing concentrations of isoflurane caused a mild dose-related metabolic acidosis | - BS measured by visualization limiting accuracy - Baseline values before use of isoflurane not reported, limiting conclusions that can be drawn - CVR not measured but calculated indirectly. |
| Roald et al., 1991 (30) | Mongrel Dogs | In a group of dogs, isoelectric EEG was induced by isoflurane and CBF and CMR_O2_ was measured before and after reversal with nitric oxide. | 6 | Isoflurane (3.1%) | EEG visualized | Measuring blood flow in sagittal sinus  CMR_O2_= CBF X (O2_SA_-O2_SS_) | - EEG was made isoelectric with 3.1% isoflurane. With addition of nitric oxide, EEG activity increased. With 3.5% concentration of isoflurane with nitric oxide, the EEG became isoelectric again. - MAP was maintained throughout the study with angiotensin - With isoelectric EEG, there was no significant change in CBF - With isoelectric EEG, CMR_O2 ,_ decreased by 40% compared to baseline condition (1.4% isoflurane with nitric oxide). With addition of nitric oxide, CMR_O2_ was still 43% less than baseline. With increase of isoflurane concentration, CMR_O2_ was 43% less | - BS measured by visualization limiting accuracy - No baseline measurement of CBF without isoflurane - Low sample size |
| Scheller et al., 1986 (31) | New Zealand White Rabbits | In a group of 18 rabbits BS was induced and CBF in the cortex, white matter, and hippocampus as well as total ICP were measured at three different concentrations of CO_2_. | 18 | Isoflurane (2.05%) | EEG visualized | Hydrogen clearance technique | - At a concentration of 1.0 MAC, isoflurane induced deep BS. - Administration of isoflurane did not lead to any significant changes in MAP which was maintained by angiotensin - During normocapnia, there was no change in CBF with BS. In the hypercapnia condition, there was a 79% significant (p<0.05) increase in CBF in the dorsal hippocampus with BS. In the hypocapnia condition, there was a 43% significant decrease in CBF in the cortex, 38% decrease in the white matter, and 37% decrease in the dorsal hippocampus (p<0.05) with BS. - Induction of BS lead to a 183% significant increase in ICP in the hypocapnia condition (p<0.05), 94% significant increase in the normocapnia condition (p<0.05), and a 270% increase in the hypercapnia condition (p<0.05) | - Animals already anaesthetized with nitric oxide at baseline which is known to nullify effect of isoflurane, confounding comparison - CBF measured indirectly, limiting accuracy - BS measured by visualization limiting accuracy |
| Sirmpilatze et al., 2022 (32) | Non-human primates (macaques/marmosets) and Wistar Rats | In groups of macaques, marmosets, and rats, different concentrations of sevoflurane were used to suppress EEG activity to different levels and resulting BOLD signal was measured. | 13/20 and 11 | Isoflurane (0.95-2.5%) | EEG visualized and signal analysis | fMRI BOLD signal analysis | - In macaques, the striatum and most of the cortex were significantly correlated with BS (p<0.05). The cerebellum, the primary visual cortex, parts of somatosensory and motor cortices on either side of the central sulcus, the subcallosal cortex, and the parahippocampal gyrus were not significantly correlated. No significant anticorrelations were found. - The marmoset brain correlation map was similar to the macaque map - In rats, all primary motor and sensory cortices were correlated with BS (p<0.05) whereas the cerebellum amygdala did not. Parts of the thalamus and hippocampus were also significantly correlated. | - CBF measured indirectly, limiting accuracy and conclusions that can be drawn. - Conclusions cannot be drawn on the effect of BS on overall, average CBF |
| Sutin et al., 2014 (33) | Sprague Dawley Rats | In a group of rats, BS and isoelectric EEG were induced and variation in CBF and CMR_O2_ measured. | 10 | Isoflurane (1%-3%) | EEG Visualized | Near Infrared Spectroscopy  Optical Diffusion Correlation Spectroscopy | - At 2% isoflurane, there was BS, and spikes in CBF and CMR_O2_ match spikes in EEG. EEG spikes occur more rapidly than CBF recovery, leading to overlapping hemodynamic responses. - At 3%, EEG is isoelectric and there is little variation in CBF and CMR_O2_ | - No quantification of average CBF at baseline and post-BS, limiting conclusions that can be drawn - No assessment of significance - BS measured by visualization, limiting accuracy |
| Walter et al., 2013 (34) | Juvenile and Neonatal pigs | In a group of neonatal and in a separate group of juvenile pigs, CBF, CMR_O2_, and CMR_glu_ were measured under light sedation and under BS with separate techniques. | 14/11 | Isoflurane (0.25%) | (Intracranial) EEG power spectral analysis | FDG-PET  CMR_O2_= CBF X (O2_SA_-O2_SS_) | - There was no significant change in MAP with onset of BS - In both neonatal and juvenile pigs, there was a significant reduction in CBF 10 minutes post onset of BS (48%/46%; p<0.05) as well as 75 minutes after onset of BS (43%/44%; p<0.05) - In both juvenile and neonatal pigs, there was a significant reduction in CMR_O2_ 10 minutes post onset of BS (42%/31%; p<0.05) and as well as 75 minutes after onset of BS (33%/31%; p<0.05) - In both juvenile and neonatal pigs, there was a significant reduction in CMR_glu_ 10 minutes post onset of BS (45%/50%; p<0.05) and as well as 75 minutes after onset of BS (42%/48%; p<0.05) - FDG-PET results showed that, during BS, there was significant reduction in metabolism for glucose in the neocortex (−31%), basal ganglia (−25%), and thalamus (−20%) in neonatal pigs and a reduction in all brain regions (-24% - -41%) in juvenile pigs (p<0.05) | - CBF measured indirectly, limiting accuracy. - In baseline condition, subjects still given isoflurane confounding baseline CBF to which subsequent measurements were compared |
| Yang et al., 2018 (35) | Non-human primates | In a group of primates, BS was induced and ICP and CBF measured. | Unspecified | Isoflurane (1-3%) | EEG Visualized | Near Infrared Spectroscopy  ICP measurement | - There was no significant change in MAP with onset of BS - After onset of BS, ICP increased by 40% and total Hgb decreased by 0.2-0.3 µM followed by a 0.5 µM increase. - The change in ICP seemed to be time-locked to the change in CBF | - Sample size not specified - No assessment of significance - CBF measured indirectly, limiting accuracy |
| Zhang et al., 2019 (36) | Non-human primates | In a group of nonhuman primates, BS was induced and BOLD signal and functional connectivity assessed. | 16 | Isoflurane (0.80-1.30%) | EEG BS ratio | fMRI and BOLD signal | - BS only seen in 2 of the primates - There was strong coupling and a positive correlation between BOLD signals and EEG burst signals (p<0.001). This was associated with enhancement of whole brain connectivity, especially in the thalamocortical networks | - Low sample size in which BS was induced - BOLD signal examines CBF indirectly, limiting accuracy - Conclusions that can be drawn from functional connectivity analysis in relation to CBF are limited |
| Zornow et al., 1990 (37) | Mongrel dogs | In a group of dogs, BS was induced by isoflurane and CBF and CMR_O2_ were measured. | 6 | Isoflurane | EEG visualized | Measuring blood flow in sagittal sinus  CMR_O2_= CBF X (O2_SA_-O2_SS_) | - BS was achieved with 2.2 MAC of isoflurane - CBF decreased by 19% (0.01<p<0.05) when at 2.2 MAC concentration compared with 0.5 MAC - CMR_O2_ was significantly 42% less when at 2.2 MAC concentration compared with 0.5 MAC (p<0.01) | - No baseline measurement of CBF without isoflurane - BS measured by visualization limiting accuracy - Low sample size |
| Multiple |  |  |  |  |  |  |  |  |
| Harper and Mackenzie, 1977 (38) | Healthy Baboons | In a group of 7 health baboons, BS was induced and the effects of the addition of 5HT and urea on CBF, CMR_O2_ and CMR_glu_ was investigated. | 7 | Sodium thiopentone,phencyclidine, nitrous oxide | EEG visualized | Xenon clearance method  CMR_O2_= CBF X (O2_SA_-O2_SS_)  CMR_glu_= CBF x (Glu_art_- Glu_v_) | - MAP stayed constant - 5HT caused significant constriction of internal carotid artery - At baseline, with phencyclidine and nitrous oxide, continuous activity/mild BS was seen. After addition of 5HT and urea, burst-suppression/isoelectricity was seen - Compared to baseline condition, there was a 29% significant decrease in CBF (p<0.01), 35% decrease in CMR_O2_ (p<0.01), and 35% decrease in CMR_glu_ (p<0.05) in the 5HT + urea condition x | - BS measured by visualization, limiting accuracy - There is no baseline measurement of CBF prior to anaesthesia - 5HT caused vasoconstriction of the carotid artery in addition to affecting BS, limiting the conclusions that can be drawn. |
| Joshi et al., 2004(39) | New Zealand White Rabbits | In a group of 16 rabbits, isoelectric EEG was induced and CBF and CVR was measured with thiopental, with propofol, and with saline before and after carotid occlusion. | 16 | Intracarotid Thiopental (1%) and propofol (1%) | EEG Visualized | Laser doppler flowmetry | - In all 3 groups, before carotid occlusion, there was no significant difference in CBF or CVR. - In all 3 groups, CBF significantly decreased with carotid artery occlusion - During reperfusion, when propofol and thiopental was injected, CBF showed significant increase in all 3 groups but again, there was no difference between the groups | - Isoelectricity/BS measured by visualization, limiting accuracy - Isoelectric EEG not monitored continuously; dose determined before experiment - No measurement of CMR_O2_ limiting conclusions that can be drawn |
| Warner et al., 1989 (40) | Sprague-Dawley Rats | In a group of rats, BS was induced by pentobarbital and by isoflurane (separate groups) and change in CBF and CMR_glu_ was compared between the conditions at baseline and after MCA occlusion. | 10 | IV Pentobarbital and Isoflurane (1.9-2.1%) | EEG visualized | Quantitative autoradiography | - There was no significant change in MAP in the pentobarbital or isoflurane conditions - Compared with the sham rat, rats in the pentobarbital condition had a 67% lower CBF while there was no change in the CBF in the isoflurane rats. The difference in CBF between the isoflurane and pentobarbital groups was significant (p<0.001) but there was no comparison between sham rats and drug rats. - MCA occlusion led to significantly decreased blood flow in all regions but was 100% greater in isoflurane anesthetized rats compared to pentobarbital anaesthetized rats (p<0.001) - There was no significant difference in CMR_glu_ between the isoflurane and pentobarbital conditions | - BS measured by visualization limiting accuracy - CBF measured indirectly, limiting accuracy - Baseline CBF and CMR_glu_ can only be inferred from sham conditions and no comparison between sham rats and drug rats, limiting conclusions that can be drawn - Plasma glucose significantly elevated in isoflurane condition compared to pentobarbital condition confounding comparison in CMR_glu_ |
| Young et al., 1997 (41) | Sprague-Dawley Rats | In a group of rats, BS was induced by propofol or isoflurane and CBF and CMR_O2_ were inferred by measurement of infarct volume after injury was induced by ischemia-reperfusion model. | 20 | Propofol 1mg/kg/min  OR  Isoflurane 3% | EEG Visualized | Measurement of infarct volume | - There was no significant difference between MAP in the propofol group or MAP in the isoflurane group - The propofol group showed a 21% reduction in percentage infarct volume compared with the isoflurane group (p<0.001) | - No measurement of CBF or CMR_O2_ so limits conclusions that can be drawn - No baseline measurement prior to induction of BS, limiting conclusions that can be drawn - BS was measured by visualization, limiting accuracy |
| Other |  |  |  |  |  |  |  |  |
| Werner et al., 1995 (42) | Mongrel Dogs | In a group of dogs, BS was induced by hemorrhagic hypotension and CBF velocity, ICP, and CPP were recorded. | 11 | Hemorrhagic Hypotension | EEG Power Analysis | TCD of MCA  ICP measured with catheter inside lateral ventricle  CPP = MAP - ICP | - The occurrence of BS EEG with isoelectric periods of more than 3 s was considered the cerebral ischemic threshold - EEG BS occurred at a MAP of 31 +/- 7 mmHg. At this MAP, there was no significant change in ICP but CPP was significantly reduced by 72% (p<0.05). TCD diastolic velocities were 0 cm/s and systolic velocities were 10 cm/s (compared with 70cm/s and 50 cm/s at baseline) - At a MAP below this level, ICP was significantly reduced by 50% and CPP was significantly reduced by 78% (p<0.05) | - Experimental design itself affects CBF, limiting conclusions that can be drawn |

Legend:

BS=Burst Suppression

MAP= Mean Arterial pressure

CBF= Cerebral Blood Flow

ICP=intracranial pressure

CPP= cerebral perfusion pressure

CVR=Cerebral vascular resistance

CMR_O2_= cerebral metabolic rate for oxygen

CBF X (O2_SA_-O2_SS_)

EEG= Electroencephalogram

fMRI= functional magnetic resonance imaging

BOLD= blood oxygen level dependent

NADH= nicotinamide adenine dinucleotide (NAD) + hydrogen (H)

CF=Cerebral flow

O2_SA_= O2 in systemic circulation

O2_SS_=O2 in sagittal sinus

O2_JV_=O2 in jugular vein

C(a-v)O2= Cerebral Arterial-Venous Difference in Oxygen

SSP= superior sagittal sinus pressure

MAC= minimum alveolar concentration

TCD=transcranial doppler

PI=Pulsatility index

V_systolic_= CBF velocity during systole

V_diastole_= CBF velocity during diastole

CMR_glu_ = cerebral metabolic rate for glucose

lCMR_glu_ = local cerebral metabolic rate for glucose

ROI: Region of interest (for BOLD signal analysis)

Glu_art_= Arterial glucose

Glu_v_ = Venous glucose

MCA=middle cerebral artery

Hgb=hemoglobin

deoxyHgb= deoxygenated hemoglobin

oxyHgb= oxygenated hemoglobin

SaO2=systemic arterial oxygen concentration

MR= Magnetic resonance

CTOP= cortical tissue oxygen pressure

FDG-PET= fluorodeoxyglucose (FDG)-positron emission tomography

5HT= Serotonin

**Supplementary Table 2:** **Summary of articles examining the effect of BS on CBF in humans.**

| **Article** | **Study Subjects** | **Experimental Conditions** | **Number of Subjects** | **Method of BS** | **BS Determination** | **Measure of CBF** | **Study Results and Conclusions** | **Study Limitations** |
| --- | --- | --- | --- | --- | --- | --- | --- | --- |
| Propofol |  | | | | | | | |
| Chaix et al., 2019 (43) | Adult patients undergoing neuroradiological procedures | A group of patients undergoing neuroradiological procedure were prospectively observed and changes in CBF velocity in response to propofol were recorded. | 81 | Propofol | EEG Spectral Analysis | Transcranial doppler  Near infrared spectroscopy | - Norepinephrine was used to maintain MAP >65mmHg - Patients were divided into “high-risk” (>1 vascular risk factor) and “low-risk” ( 1 vascular risk factor or lower) groups. In the low risk group, 11% developed BS, and in the high risk group 57% developed BS - With anaesthesia induction, there was a mean 34% decrease in MAP in the high risk group and mean 17% decrease in the low risk patients (p<0.001). - With anaesthesia induction, there was a 39% decrease in CBF velocity in the high risk group and mean 28% decrease in CBF velocity in the low risk group (p<0.01) - When norepinephrine was used to increase MAP, there was a higher increase in CBF velocity in the high risk group vs the low risk group (15% vs 4%, p<0.01) | - Prospective observational study so level of evidence low - Not all patients were burst suppressed; different amounts of burst suppressed patients per group |
| Doyle and Matta, 1999 (44) | Adult patients undergoing vestibular schwannoma resection | In a group of patients undergoing surgery, CBF velocity and AVD_O2_ was measured at different BS ratios. | 9 | Propofol 6-10 mg/kg/hr | EEG Visualized | TCD  Jugular bulb oxygen saturation monitoring | - Phenylephrine was used to maintain MAP within 20% of baseline - With increasing BS ratio, CBF velocity decreased; 24% with 50% BS ratio, 37% with 100% BS ratio (isoelectricity; p<0.05) - There was no significant change in AVD_O2_ | - BS measured by visualization limiting accuracy |
| Klein et al., 2011 (45) | Adult patients undergoing elective neurosurgical procedures | In a group of patients undergoing surgery, EEG was suppressed and resulting venous flow velocity, AVD_O2_, and CMR_O2_ was measured under different levels of EEG suppression. | 21 | Propofol 4-10 mg/kg/hr | EEG spectral analysis | Laser doppler flowmetry  CMR_O2_= CBF x AVD_O2_ | - MAP was maintained with cafedrine-hcl and theodrenaline-hcl. - Propofol concentration was adjusted to achieve higher bispectral index (target 40) or lower bispectral index (target 20). - AVD_O2_ was approximately 25% lower (p=0.025) and CMR_O2_ was approximately 58% lower (p=0.022) in the lower BIS group - There was no significant change in venous capillary blood flow | - Exact values of AVD_O2_ and CMR_O2_ not given - Unclear if BS was achieved - Arterial CBF not measured |
| Ludbrook et al., 2002 (46) | Adult patients undergoing orthopedic procedures | In a group of patients undergoing surgery, BS was induced and CBF velocity and AVD_O2_ was measured at different times of infusion. | 7 | Propofol 110mg/min and 10mg/min | EEG spectral analysis | TCD  Jugular bulb oxygen saturation monitoring | - MAP not maintained with pressors and dropped by about 25% in the first 5 minutes of infusion - BS was induced between 6.5 and 7.5 min and had almost completely recovered by 35 min - CBF velocity reached a minimum of 42% of baseline, 6 minutes from onset of propofol (p<0.0001) - There was no significant change in AVD_O2_ | - Unclear if MAP drop was significant - Challenging to draw conclusions about the effect of BS on CBF velocity |
| Matta et al., 1995 (47) | Adult patients undergoing non-neurological surgery | In a group of patients undergoing surgery, isoelectric EEG was induced by propofol, and CA and carbon dioxide reactivity measured by increasing MAP with phenylephrine and measuring the resultant CBF velocity in the MCA | 10 | Propofol 2.5mg/kg | EEG visualized | TCD  CVR=MAP/CBF velocity in MCA | - MAP was maintained within the range of 70-90 mmHg with phenylephrine - With induction of BS, there was a decrease in CBF velocity by around 15% - An increase in MAP of mean 24mmHg had no effect on CBF velocity or relative carbon dioxide reactivity | - CBF velocity only reported visually and no significance value reported - BS measured by visualization limiting accuracy |
| Newman et al., 1995 (48) | Adult patients undergoing elective cardiac valvular surgery | In a group of adult patients undergoing cardiac surgery, CBF, CMR_O2_, AVD_O2_, and O2_JV_ were measured in 15 control patients received sufentanil and vecuronium and 15 patients received burst suppressive doses of propofol during normothermia, hypothermia, and a return to normothermia. | 30 | Propofol | EEG Visualized | Xenon clearance method  Jugular bulb oxygen saturation monitoring  CMR_O2_= CBF X (O2_SA_-O2_JV_) | - MAP was maintained between 50 and 90 mmHg with phenylephrine or sodium nitroprusside. There was no significant difference in MAP between groups - In the normothermia condition, in the propofol group, CBF was 44% significantly lower compared to controls (p<0.05) - In the hypothermia condition, in the propofol group, CBF was 41% significantly lower compared to controls (p<0.05) - When the hypothermia group was warmed to normothermia, in the propofol group, CBF was 37% significantly lower compared to controls (p<0.05) - CMR_O2_ followed the same pattern, being significantly lower in all conditions in the propofol group vs the control group - There was no significant difference in AVD_O2_ or in O2_JV_ between groups. | - No baseline measurements in propofol group before induction of BS, limiting conclusions that can be drawn - BS measured by visualization, limiting accuracy - CBF measured indirectly using xenon clearance, limiting accuracy |
| Barbiturates |  | | | | | | | |
| Bendtsen et al., 1985 (49) | Adult patients undergoing craniotomy for cerebral tumors | In a group of patients with supratentorial brain tumors, BS was induced by Althesin and CBF and CMR_O2_ measured at different levels of EEG suppression. | 10 | Althesin 0.2 ml/kg/hr | EEG Observed | Xenon clearance method  Jugular bulb oxygen saturation monitoring  CMR_O2_= CBF X (O2_SA_-O2_JV_) | - Unclear if MAP was maintained with pressors - 6 patients reached BS with Althesin 0.2ml/kg/hr - The CBF was variable between patients, ranging from 15 to 28 mL/min/100g. - In patients who did not reach BS, the CBF ranged from 19 to 31 mL/min/100g - In 3 patients a decrease in CMR_O2_ was associated with suppression of EEG activity. In 1 patient, the EEG was suppressed without any decrease in CMR_O2_. In 6 patients, there was a decrease in CMR_O2_ without any suppression of EEG. | - Low sample size - There was no baseline measurement of CBF in individual patients, limiting conclusions that can be drawn - CBF is recorded at different levels of suppression, limiting conclusions that can be drawn - No combined analysis of all patient data, limiting conclusions that can be drawn - Xenon clearance method is indirect measure of CBF, limiting accuracy |
| Connolly et al., 2015 (50) | Adult critically ill patients | In one patient with traumatic brain injury and another with aneurysmal subarachnoid hemorrhage, BS was achieved and resulting change in ICP and VDI measured and correlated to the bursts. | 2 | Pentobarbital | EEG Observed | ICP monitoring and vasodilation index (VDI) obtained from ICP pulse waveform | - Unclear if MAP was maintained with pressors - 79.6% of bursts were associated with an increase in ICP - The mean change in ICP amplitude between the burst onset and the peak or onset of the next burst was 0.54 mmHg - The median VDI for the increasing ICP segments was 0.56 - The magnitude of the VDI correlated with burst duration (correlation coefficient = 0.2, p<0.001) | - Small sample size - BS measured by visualization, limiting accuracy - No comparison to baseline, non-burst suppressed state - No direct assessment of CBF or CMR_O2_ |
| Hoffman et al., 1997 (51) | Adult  patients undergoing craniotomy for cerebral aneurysms | In a group of patients undergoing surgery for aneurysms, BS was induced by etomidate and the effects on CBF and CMR_O2_ measured in the same patients. | 7 | Etomidate 125 μg/kg/min | EEG Visualized | Flow probe method  Jugular bulb oxygen saturation monitoring  CMR_O2_= CBF X (O2_SA_-O2_JV_) | - Unclear if MAP was maintained with pressors - When BS was attained, there was a significant 10% decrease in MAP (p<0.05) - When BS was attained, there was a significant 40% reduction in CBF and CMR_O2_ (p<0.05) | - Unclear if C(a-v) O2 or O2_JV_ changed with induction of BS - Significant decrease in MAP between conditions limits conclusions that can be drawn - BS measured by visualization limiting accuracy |
| Young et al., 1991 (52) | Adult patients undergoing carotid endarterectomy | In a group of patients undergoing carotid endarterectomy, anaesthesia was induced with isoflurane and BS achieved by thiopental and CBF measured. | 5 | Thiopental 2.6-5.8mg/kg | EEG Visualized | Xenon clearance method | - Two of the patients received phenylephrine so that there was no mean significant change in MAP - When BS was achieved, CBF decreased by 48% (p<0.01) - There were no hemispheric differences in change in CBF | - Low sample size - CMR_O2_ not assessed - Baseline anaesthesia used was isoflurane, confounding baseline measurement - CBF measured indirectly limiting accuracy - BS measured by visualization limiting accuracy |
| Inhalational |  |  |  |  |  |  |  |  |
| Artru et al., 1997 (53) | Adult patients undergoing elective neurosurgery | In a group of patients undergoing surgery, BS was induced with different concentrations of sevoflurane and isoflurane and CBF velocity and CPP measured. | 14 (8 sevoflurane, 6 isoflurane) | 0.5-1.5 MAC  Isoflurane (1-3%)  Sevoflurane (0.5-1.5%) | EEG visualized | TCD | - No pressor was used to maintain MAP, concentration of anesthetics were adjusted accordingly. - At 1.5 MAC, a BS pattern was predominant in 2 patients receiving sevoflurane and 1 patient receiving isoflurane. - At 1.5 MAC isoflurane, there was a 28% significant reduction in MAP, 36% significant reduction in CPP, 30% reduction in CBF velocity and no significant reduction in CVR (p<0.05) - At 1.5 MAC sevoflurane, there was no significant reduction in MAP or CPP. There was a 32% significant reduction in CBF velocity and 41% significant increase in CVR (p<0.05) | - Low number of patients achieved BS - BS measured by visualization limiting accuracy - CMR_O2_ not measured |
| Golkowski et al., 2017 (54) | Healthy adults | In a group of healthy adults, BS as induced and CBF was measured by measuring BOLD signal | 19 | Sevoflurane 3% | EEG power analysis | BOLD signal analysis | - Norepinephrine was used to maintain MAP close to baseline values - BS was achieved in all patients - Compared with the model overall hemodynamic response (amplitude 4.44% and time to peak 13s), the sevoflurane response had a lower amplitude (1.14% and time to peak 26s) - There was significant correlation of BOLD signal with BS in frontal, parietal, and temporal lobes and in basal ganglia (p<0.001). BOLD signal increased after burst onset and decrease after EEG suppression. - In the occipital lobe, BOLD signal was anticorrelated with BS (p<0.001). | - CBF measured indirectly, limiting accuracy and conclusions that can be drawn |
| Lam et al., 1995 (55) | Adult patients undergoing peripheral orthopedic surgery | In a group of adult patients undergoing surgery, isoelectric EEG was induced and CBF velocity measured. | 8 | 1.5-2.0 MAC  Isoflurane  Desflurane | EEG visualized | TCD | - Phenylephrine used to maintain MAP above 60 mmHg - With the onset of electrical silence in EEG, the CBF velocity decreased by 38% (p<0.001). Reappearance of EEG was associated with sudden increase in CBF velocity. | - No assessment of CMR_O2_ - BS measured by visualization, limiting accuracy - No comparison between anaesthetics |
| Reinsfelt et al., 2003 (56) | Adult patients undergoing coronary bypass graft or valve surgery | In a group of patients undergoing surgery at hypothermic temperatures (32°C), BS was induced and MAP changed by the use of phenylephrine, and CBF velocity, CPP, and AVD_O2_ measured. | 16 | Isoflurane 1.5% | EEG Visualized | TCD  Jugular bulb oxygen saturation monitoring  Cerebral oxygen extraction (COE)= AVD_O2_/SaO2 | - MAP was manipulated using sodium nitroprusside and norepinephrine - There was a 27% significant decrease in CBF velocity (p<0.05) - COE decreased significantly by 13% (p<0.05) - Under BS, there was a steeper positive slope of relationship between CBF velocity and CPP and a steeper negative slope between CPP and COE (p<0.05) | - BS measured by visualization, limiting accuracy - At baseline, patients anaesthetized with propofol, limiting conclusions that can be drawn |
| Reinsfelt et al., 2011 (57) | Adult patients undergoing coronary bypass graft or valve surgery | In a group of patients undergoing surgery at hypothermic temperatures (32°C), BS was induced and MAP changed by the use of phenylephrine, and CBF velocity, CPP, and AVD_O2_ measured. | 16 | Sevoflurane 3.36% | EEG Visualized | TCD  Jugular bulb oxygen saturation monitoring  Cerebral oxygen extraction (COE)= AVD_O2_/SaO2 | - MAP was manipulated using sodium nitroprusside and norepinephrine - There was a 17% significant decrease in CBF velocity (p<0.05) - COE decreased significantly by 22% (p<0.05) - Under BS, there was a steeper positive slope of relationship between CBF velocity and CPP and a steeper negative slope between CPP and COE (p<0.01) | - BS measured by visualization, limiting accuracy - At baseline, patients anaesthetized with propofol, limiting conclusions that can be drawn |
| Sirmpilatze et al., 2022 (32) | Healthy adults | In a group of healthy adults, different concentrations of sevoflurane were used to suppress EEG activity to different levels and resulting BOLD signal was measured | 20 | Sevoflurane 3.9–4.6% | EEG Visualized and signal analysis | fMRI BOLD signal analysis | - Unclear if pressors were used to maintain MAP goals - 19/20 participants attained BS in the high sevoflurane concentration group - There was more widespread fluctuation in the cortical BOLD signal during BS - Cortical sensory areas and anterior and midline parts of the thalamus were significantly correlated with burst-suppression (p<0.05). The posterior thalamic nuclei, the cerebellar cortex, the hippocampus, and the amygdala were not. - Regions adjacent to the ventricular borders were anticorrelated with BS | - CBF measured indirectly, limiting accuracy and the conclusions that could be drawn - Cannot draw conclusions about the effect of BS on overall, average CBF |
| Multiple |  |  |  |  |  |  |  |  |
| Akbik et al., 2020 (58) | Adult critically ill patients | In a group of neurologically critically ill patients, BS was induced by different methods and FDG uptake measured. | 6 | Propofol  Pentobarbital  Midazolam | EEG Visualized | FDG-PET | - Unclear if pressors were used to maintain MAP goals - In 4 patients with status epilepticus, stroke-like migraine attacks after radiotherapy, and epilepsy secondary to cerebral amyloid angiopathy, FDG uptake in lesional and non-lesional areas decreased after BS. - In two patients with viral or autoimmune encephalitis, FED uptake was increase in lesional and non lesional areas after BS. | - FDG uptake not quantified - Low sample size - Heterogenous group of patients - Heterogeneous methods of BS induction - No combined analysis - CBF measured indirectly, limiting accuracy and conclusions that can be drawn - BS measured with visualization limiting accuracy |
| Matta et al., 1995 (59) | Adult patients undergoing peripheral orthopedic surgery | In a group of patients undergoing surgery, BS was induced by propofol and CBF velocity measured with exposure to different inhalation anaesthetics. | 21 | Propofol 2.5-3mg/kg  0.5 and 1.5 MAC and  Halothane (0.76%)  Isoflurane (1.15%)  Desflurane (6.0%) | EEG visualized | TCD  Jugular bulb oxygen saturation monitoring  CBFe=1/AVD_O2_ | - Phenylephrine used to maintain MAP between 70 and 90 mmHg - When EEG suppression was attained with propofol, there was no significant difference in CBF velocity between three groups - At 0.5MAC, halothane increased CBF velocity by 21%, isoflurane by 23% and desflurane by 21% (p<0.05) - At 1.5MAC, halothane increased CBF velocity by 50%, isoflurane by 73% and desflurane by 76% (p<0.05) - At 0.5MAC, halothane increased CBFe by 18%, isoflurane by 24% and desflurane by 23% (p<0.05) - At 1.5MAC, halothane increased CBFe by 48%, isoflurane by 73% and desflurane by 79% (p<0.05) | - Pre BS CBF velocity not reported, limiting the conclusions that can be drawn - BS measured by visualization, limiting accuracy |
| Matta and Lam, 1995 (60) | Adult patients undergoing non-neurological surgery | In a group of patients undergoing surgery, BS was induced by propofol and CBF and CMR_O2_ measured after addition of nitric oxide | 10 | Propofol 2.5mg/kg  Nitric Oxide | EEG Visualized | TCD  CVR=MAP/CBF velocity in MCA  Jugular bulb oxygen saturation monitoring | - Phenylephrine was used to maintain MAP between 70 and 90 mmHg - With addition of nitric oxide, CBF velocity increased by 21% (p<0.01) but there was no significant change in AVD_O2_ - With addition of nitric oxide, there was a decrease in CVR by 16% (p<0.05) and an increase in CMR_O2_ by 14% | - Pre-BS CBF velocity not reported, limiting conclusions that can be drawn - BS measured by visualization, limiting accuracy |
| Matta et al., 1999 (61) | Adult patients undergoing spine surgery | In a group of adult patients undergoing surgery, isoelectric EEG was induced by propofol and the effect of inhalational anaesthetics on CBF measured. | 20 | Propofol 0.26 mg/kg/min  Sevoflurane 0.5-1.5 MAC  Isoflurane 0.5-1.5 MAC | EEG Visualized | TCD | - Isoelectric EEG achieved at baseline with propofol - There was no significant change in MAP with addition of inhalational anaesthetics and it was maintained by phenylephrine - With addition of 0.5 MAC sevoflurane there was a 4% increase in mean CBF velocity in the MCA and with 0.5MAC isoflurane there was a 19% increase (p<0.05) - With addition of 1.5 MAC sevoflurane there was a 17% increase in mean CBF velocity in the MCA and with 0.5 MAC isoflurane there was a 72% increase (p<0.05) | - Isoelectric EEG measured by visualization limiting accuracy - Baseline measurements taken at isoelectricity, limiting conclusions that can be drawn |
| Woodcock et al., 1987 (62) | Adult patients undergoing coronary bypass graft surgery | In a group of patients undergoing heart surgery, BS was induced by thiopental and isoflurane and CBF, CMR_O2_, and CPP were compared between groups and at different time periods of hypothermia. | 31 | Thiopental 8mg/kg  And Isoflurane | EEG Visualized | Xenon clearance method  CMR_O2_= CBF X (O2_SA_-O2_JV_)  CPP=MAP-JVP | - There was no significant difference in MAP between isoflurane, thiopental, and control groups pre-hypothermia. There was a significant decrease in MAP in isoflurane and thiopental groups after 15 minutes of hypothermia (p<0.05) - Phenylephrine was used significantly more to maintain MAP in isoflurane group compared to the thiopental or control groups (p<0.0005) and administration of vasodilators was used more commonly in the control group versus isoflurane or thiopental groups (p<0.0005) - BS was seen in thiopental and isoflurane groups but not control group - After 15 minutes of hypothermia, the CBF was 44% lower in the thiopental group compared to control (p<0.05). There was no significant difference in the CBF in the isoflurane group. - In the normothermic condition (temp 37.3), CBF in the thiopental group was 24% lower compared to control (p<0.05). There was no significant difference in the isoflurane group. - After 15 minutes of hypothermia, CMR_O2_ was 34% lower in the thiopental group and 29% lower in the isoflurane group compared to control (p<0.05). - In the normothermic condition (temp 37.3), CMR_O2_ was 21% lower in the thiopental group and 34% lower in the isoflurane group was compared to control (p<0.05). | - CBF measured indirectly, limiting accuracy - BS measured by visualization limiting accuracy. |
| Zanatta et al., 2013 (63) | Adult patients undergoing cardiopulmonary bypass surgery | In a retrospective review of adult patients undergoing surgery, multiple methods of anaesthesia were used to induce BS and resulting CBF velocity oscillations measured and correlated to bursts of activity on the EEG. | 28 | Propofol 2-4 mg/kg/min  Isoflurane 1.5 MAC | EEG Power analysis | TCD | - MAP was maintained with bypass machine - With deep sedation BS, intensity of CBF velocity oscillations decreased by 40% compared to the moderate anaesthesia condition - The BS pattern was highly correlative to the CBF velocity oscillations (R=0.849, p<0.01) | - Mean value of CBF velocity not given - No comparison of mean CBF velocity to baseline, non-anaesthetized state - CMR_O2_ not measured |
| Brain Injury |  |  |  |  |  |  |  |  |
| Chalia et al., 2016 (64) | Infants >34 weeks with hypoxic ischemic encephalopathy | In a group of infants, spikes during BS were correlated with a hemodynamic response function. | 6 | Brain Injury | EEG Visualized | Diffuse Optical Tomography | - There was a pronounced decrease in oxygenated hemoglobin just before or during EEG burst activity followed by large increase reaching peak 20s after burst onset. - Unclear if pressors were used to maintain MAP goals | - Low sample size - Global CBF not recorded - BS already at baseline, limiting conclusions that can be drawn |
| Du et al., 2014 (65) | Adult patients in medically stable, vegetative state | In a group of patients with brain contrecoup contusion, primary brain stem injury, space-occupying brain compression injury, or secondary brain stem injury, zolpidem 10mg was given and resulting BS (BS), cerebral state index (CSI), and CBF measured. | 127 | Brain injury | Cerebral state index | SPECT | - Unclear if pressors were used to maintain MAP goals - One hour after treatment with zolpidem, in the brain contrecoup contusion group, CSI was increased by 7.6% and BS decreased by 40% (p<0.05). In the space occupying brain compression group, CSI was increased by 9.1% and BS reduced by 47% (p<0.05) - Visual analysis of cerebral perfusion showed that CBF in damaged areas in brain contrecoup contusion group and space occupying brain compression injury group was increased one hour after administration of zolpidem - (CSR Z=3.55; BS Z=3.07) and space-occupying brain compression (CSI=3.63; BS=3.52) groups (p<0.05). There was no difference in CSI and BS in the other groups. | - CBF change not quantified - Given BS already at baseline, conclusions that can be draw are limited |
| Kassab et al., 2021 (66) | Adult neurologically critically patients | In a group of patients with status epilepticus or generalized encephalopathy, the correlation between EEG and hemodynamic response function was measured. | 11 | Brain Injury | EEG Visualized | Functional near-infrared spectroscopy | - In patients with status epilepticus, the representative hemodynamic response functions to bursts of spikes showed large increases in oxygenated hemoglobin and total hemoglobin - In some patients inotropes were used to maintain MAP. - There was positive correlation between seizure duration on EEG and hemodynamic response function (oxygenated hemoglobin p=0.21 R^2^=0.569, total hemoglobin p=0.034 R^2^=0.403) - BS occurred in four patients. - There were increased in oxygenated hemoglobin and total hemoglobin following onset of bursts in representative hemodynamic response functions. Duration and amplitudes of oxygenated hemoglobin were positively correlated with lengths of bursts (p=0.00017) | - Low sample size in whom BS was induced - Overall CBF not measured, limiting conclusions that can be drawn - BS measured by visualization, limiting accuracy |

Legend:

BS=Burst suppression

MAP= Mean Arterial Pressure

CBF= Cerebral Blood Flow

ICP=intracranial pressure

CPP= cerebral perfusion pressure

CVR=Cerebral vascular resistance

CMR_O2_= Cerebral metabolic rate for oxygen

EEG= Electroencephalogram

fMRI= functional magnetic resonance imaging

BOLD= blood oxygen level dependent

CF=Cerebral flow

O2_SA_= O2 in systemic circulation

O2_SS_=O2 in sagittal sinus

O2_JV_=O2 in jugular vein

C(a-v)O2= Cerebral Arterial-Venous Difference in Oxygen

AVD_O2_= Cerebral Arterial-Venous Difference in Oxygen

ROI: Region of interest (for BOLD signal analysis)

MCA=middle cerebral artery

Hgb=hemoglobin

deoxyHgb= deoxygenated hemoglobin

oxyHgb= oxygenated hemoglobin

MR= Magnetic resonance

FDG-PET= fluorodeoxyglucose (FDG)-positron emission tomography

SPECT=99mTc-ECD single-photon emission computerized tomography

1. Artru AA, Shapira Y, Bowdle TA. Electroencephalogram, cerebral metabolic, and vascular responses to propofol anesthesia in dogs. J Neurosurg Anesthesiol. 1992 Apr;4(2):99–109.

2. Joshi S, Wang M, Etu JJ, Nishanian EV, Pile-Spellman J. Cerebral blood flow affects dose requirements of intracarotid propofol for electrocerebral silence. Anesthesiology. 2006 Feb;104(2):290–8, discussion 5A.

3. Liu X, Pillay S, Li R, Vizuete JA, Pechman KR, Schmainda KM, et al. Multiphasic modification of intrinsic functional connectivity of the rat brain during increasing levels of propofol. NeuroImage. 2013 Dec;83:581–92.

4. Ramani R, Todd MM, Warner DS. A dose-response study of the influence of propofol on cerebral blood flow, metabolism and the electroencephalogram in the rabbit. J Neurosurg Anesthesiol. 1992 Apr;4(2):110–9.

5. Wang M, Agarwal S, Mayevsky A, Joshi S. Optically measured NADH concentrations are unaffected by propofol induced EEG silence during transient cerebral hypoperfusion in anesthetized rabbits. Brain Res. 2011 Jun 17;1396:69–76.

6. Werner C, Hoffman WE, Kochs E, Albrecht RF, Am Esch JS. The effects of propofol on cerebral blood flow in correlation to cerebral blood flow velocity in dogs. J Neurosurg Anesthesiol. 1992 Jan;4(1):41–6.

7. Gronert GA, Michenfelder JD, Sharbrough FW, Milde JH. Canine cerebral metabolic tolerance during 24 hours deep pentobarbital anesthesia. Anesthesiology. 1981 Aug;55(2):110–3.

8. Hungerhuber E, Zausinger S, Westermaier T, Plesnila N, Schmid-Elsaesser R. Simultaneous bilateral laser Doppler fluxmetry and electrophysiological recording during middle cerebral artery occlusion in rats. J Neurosci Methods. 2006 Jun 30;154(1–2):109–15.

9. Joshi S, Wang M, Etu JJ, Pile-Spellman J. Reducing cerebral blood flow increases the duration of electroencephalographic silence by intracarotid thiopental. Anesth Analg. 2005 Sep;101(3):851–8.

10. Kassell NF, Hitchon PW, Gerk MK, Sokoll MD, Hill TR. Alterations in cerebral blood flow, oxygen metabolism, and electrical activity produced by high dose sodium thiopental. Neurosurgery. 1980 Dec;7(6):598–603.

11. Klementavicius R, Nemoto EM, Yonas H. The Q10 ratio for basal cerebral metabolic rate for oxygen in rats. J Neurosurg. 1996 Sep;85(3):482–7.

12. Mäkiranta MJ, Jauhiainen JPT, Oikarinen JT, Suominen K, Tervonen O, Alahuhta S, et al. Functional magnetic resonance imaging of swine brain during change in thiopental anesthesia into EEG burst-suppression level--a preliminary study. Magma N Y N. 2002 Nov;15(1–3):27–35.

13. Michenfelder JD. The interdependency of cerebral functional and metabolic effects following massive doses of thiopental in the dog. Anesthesiology. 1974 Sep;41(3):231–6.

14. Milde LN, Milde JH, Michenfelder JD. Cerebral functional, metabolic, and hemodynamic effects of etomidate in dogs. Anesthesiology. 1985 Oct;63(4):371–7.

15. Nemoto EM, Klementavicius R, Melick JA, Yonas H. Suppression of cerebral metabolic rate for oxygen (CMRO2) by mild hypothermia compared with thiopental. J Neurosurg Anesthesiol. 1996 Jan;8(1):52–9.

16. Nemoto E. M. KR. Norepinephrine activation of basal cerebral metabolic rate for oxygen (CMRO2) during hypothennia in rats. Vol. 83. 1996.

17. Schmid-Elsaesser R, Schröder M, Zausinger S, Hungerhuber E, Baethmann A, Reulen HJ. EEG burst suppression is not necessary for maximum barbiturate protection in transient focal cerebral ischemia in the rat. J Neurol Sci. 1999 Jan 1;162(1):14–9.

18. Westermaier T, Zausinger S, Baethmann A, Steiger HJ, Schmid-Elsaesser R. No additional neuroprotection provided by barbiturate-induced burst suppression under mild hypothermic conditions in rats subjected to reversible focal ischemia. J Neurosurg. 2000 Nov;93(5):835–44.

19. Zarchin N, Guggenheimer-Furman E, Meilin S, Ornstein E, Mayevsky A. Thiopental induced cerebral protection during ischemia in gerbils. Brain Res. 1998 Jan 12;780(2):230–6.

20. Baughman VL, Hoffman WE, Thomas C, Albrecht RF, Miletich DJ. The interaction of nitrous oxide and isoflurane with incomplete cerebral ischemia in the rat. Anesthesiology. 1989 May;70(5):767–74.

21. Benveniste H, Lee H, Ding F, Sun Q, Al-Bizri E, Makaryus R, et al. Anesthesia with Dexmedetomidine and Low-dose Isoflurane Increases Solute Transport via the Glymphatic Pathway in Rat Brain When Compared with High-dose Isoflurane. Vol. 127, Anesthesiology (Hagerstown). 2017. p. 976–88.

22. Berndt N, Kovács R, Schoknecht K, Rösner J, Reiffurth C, Maechler M, et al. Low neuronal metabolism during isoflurane-induced burst suppression is related to synaptic inhibition while neurovascular coupling and mitochondrial function remain intact. J Cereb Blood Flow Metab Off J Int Soc Cereb Blood Flow Metab. 2021 Oct;41(10):2640–55.

23. Choi DH, Shin TJ, Kim S, Bae J, Cho D, Ham J, et al. Monitoring cerebral oxygenation and local field potential with a variation of isoflurane concentration in a rat model. Biomed Opt Express. 2016 Oct 1;7(10):4114–24.

24. Golanov EV, Reis DJ. Vasodilation evoked from medulla and cerebellum is coupled to bursts of cortical EEG activity in rats. Am J Physiol. 1995 Feb;268(2 Pt 2):R454-467.

25. Kochs E, Hoffman WE, Werner C, Albrecht RF, Schulte am Esch J. Cerebral blood flow velocity in relation to cerebral blood flow, cerebral metabolic rate for oxygen, and electroencephalogram analysis during isoflurane anesthesia in dogs. Anesth Analg. 1993 Jun;76(6):1222–6.

26. Liu X, Zhu XH, Zhang Y, Chen W. Neural origin of spontaneous hemodynamic fluctuations in rats under burst-suppression anesthesia condition. Cereb Cortex N Y N 1991. 2011 Feb;21(2):374–84.

27. Lutz LJ, Milde JH, Milde LN. The cerebral functional, metabolic, and hemodynamic effects of desflurane in dogs. Anesthesiology. 1990 Jul;73(1):125–31.

28. Maekawa T, Tommasino C, Shapiro HM, Keifer-Goodman J, Kohlenberger RW. Local cerebral blood flow and glucose utilization during isoflurane anesthesia in the rat. Anesthesiology. 1986 Aug;65(2):144–51.

29. Newberg LA, Milde JH, Michenfelder JD. The cerebral metabolic effects of isoflurane at and above concentrations that suppress cortical electrical activity. Anesthesiology. 1983 Jul;59(1):23–8.

30. OK R, M F, MS H, PA S. Cerebral effects of nitrous oxide when added to low and high concentrations of isoflurane in the dog. Vol. 72, Anesthesia and analgesia. Department of Anesthesiology, Aker Hospital, Oslo, Norway.; 1991. p. 75–9.

31. Scheller MS, Todd MM, Drummond JC. Isoflurane, halothane, and regional cerebral blood flow at various levels of PaCO2 in rabbits. Anesthesiology. 1986 May;64(5):598–604.

32. Sirmpilatze N, Mylius J, Ortiz-Rios M, Baudewig J, Paasonen J, Golkowski D, et al. Spatial signatures of anesthesia-induced burst-suppression differ between primates and rodents. Meng M, Makin TR, Sasai S, Faber C, editors. eLife. 2022 May 24;11:e74813.

33. Sutin J, Chang C, Boas D, Brown E, Franceschini MA. Diffuse Optical Spectroscopy Measurement Of Cerebral Hemodynamics And Oxygen Metabolism During Anesthesia-Induced Burst Suppression In Rats. In: Biomedical Optics 2014 (2014), paper BT5B3 [Internet]. Optica Publishing Group; 2014 [cited 2023 Mar 9]. p. BT5B.3. Available from: https://opg.optica.org/abstract.cfm?uri=BIOMED-2014-BT5B.3

34. Walter B, Eiselt M, Cumming P, Xiong G, Hinz R, Uthe S, et al. Resistance of brain glucose metabolism to thiopental-induced CNS depression in newborn piglets. Int J Dev Neurosci Off J Int Soc Dev Neurosci. 2013 May;31(3):157–64.

35. Yang J, Ruesch A, Schmitt S, Smith MA, Kainerstorfer JM. Correlation of EEG with Intercranial Pressure and Cerebral Hemodynamics during Burst-Supression. In: Biophotonics Congress: Biomedical Optics Congress 2018 (Microscopy/Translational/Brain/OTS) (2018), paper JTu3A40 [Internet]. Optica Publishing Group; 2018 [cited 2023 Mar 9]. p. JTu3A.40. Available from: https://opg.optica.org/abstract.cfm?uri=Translational-2018-JTu3A.40

36. Zhang Z, Cai DC, Wang Z, Zeljic K, Wang Z, Wang Y. Isoflurane-Induced Burst Suppression Increases Intrinsic Functional Connectivity of the Monkey Brain. Front Neurosci [Internet]. 2019 [cited 2023 Mar 9];13. Available from: https://www.frontiersin.org/articles/10.3389/fnins.2019.00296

37. Zornow MH, Fleischer JE, Scheller MS, Nakakimura K, Drummond JC. Dexmedetomidine, an alpha 2-adrenergic agonist, decreases cerebral blood flow in the isoflurane-anesthetized dog. Anesth Analg. 1990 Jun;70(6):624–30.

38. Harper MA, MacKenzie ET. Cerebral circulatory and metabolic effects of 5-hydroxytryptamine in anesthetized baboons. J Physiol. 1977 Oct;271(3):721–33.

39. Joshi S, Wang M, Nishanian EV, Emerson RG. Electrocerebral silence by intracarotid anesthetics does not affect early hyperemia after transient cerebral ischemia in rabbits. Anesth Analg. 2004 May;98(5):1454–9, table of contents.

40. Warner DS, Hansen TD, Vust L, Todd MM. Distribution of cerebral blood flow during deep isoflurane vs. pentobarbital anesthesia in rats with middle cerebral artery occlusion. J Neurosurg Anesthesiol. 1989 Sep 1;1(3):219–26.

41. Young Y, Menon DK, Tisavipat N, Matta BF, Jones JG. Propofol neuroprotection in a rat model of ischaemia reperfusion injury. Eur J Anaesthesiol. 1997 May;14(3):320–6.

42. Werner C, Hoffman WE, Kochs E, Albrecht RF, Schulte am Esch J. Transcranial Doppler sonography indicates critical brain perfusion during hemorrhagic hypotension in dogs. Anesth Analg. 1995 Dec;81(6):1203–7.

43. Chaix I, Manquat E, Liu N, Casadio MC, Ludes PO, Tantot A, et al. Impact of hypotension on cerebral perfusion during general anesthesia induction: A prospective observational study in adults. Acta Anaesthesiol Scand. 2020 May;64(5):592–601.

44. Doyle PW, Matta BF. Burst suppression or isoelectric encephalogram for cerebral protection: evidence from metabolic suppression studies. Br J Anaesth. 1999 Oct;83(4):580–4.

45. Klein KU, Fukui K, Schramm P, Stadie A, Fischer G, Werner C, et al. Human cerebral microcirculation and oxygen saturation during propofol-induced reduction of bispectral index. Vol. 107, British Journal of Anaesthesia. 2011. p. 735–41.

46. Ludbrook GL, Visco E, Lam AM. Propofol: relation between brain concentrations, electroencephalogram, middle cerebral artery blood flow velocity, and cerebral oxygen extraction during induction of anesthesia. Anesthesiology. 2002 Dec;97(6):1363–70.

47. Matta BF, Lam AM, Strebel S, Mayberg TS. Cerebral pressure autoregulation and carbon dioxide reactivity during propofol-induced EEG suppression. Br J Anaesth. 1995 Feb;74(2):159–63.

48. Newman MF, Murkin JM, Roach G, Croughwell ND, White WD, Clements FM, et al. Cerebral physiologic effects of burst suppression doses of propofol during nonpulsatile cardiopulmonary bypass. CNS Subgroup of McSPI. Anesth Analg. 1995 Sep;81(3):452–7.

49. Bendtsen A, Kruse A, Madsen JB, Astrup J, Rosenørn J, Blatt-Lyon B, et al. Use of a continuous infusion of althesin in neuroanaesthesia. Changes in cerebral blood flow, cerebral metabolism, the EEG and plasma alphaxalone concentration. Br J Anaesth. 1985 Apr;57(4):369–74.

50. Connolly M, Vespa P, Pouratian N, Gonzalez NR, Hu X. Characterization of the relationship between intracranial pressure and electroencephalographic monitoring in burst-suppressed patients. Neurocrit Care. 2015 Apr;22(2):212–20.

51. Hoffman WE, Charbel FT, Ausman JI. Cerebral blood flow and metabolic response to etomidate and ischemia. Neurol Res. 1997 Feb;19(1):41–4.

52. WL Y, I P, JW C, N O, E O. Thiopental effect on cerebral blood flow during carotid endarterectomy. Vol. 3, Journal of neurosurgical anesthesiology. Department of Anesthesiology, Columbia University College of Physicians and Surgeons, New York, New York, USA.; 1991. p. 265–9.

53. Artru AA, Lam AM, Johnson JO, Sperry RJ. Intracranial pressure, middle cerebral artery flow velocity, and plasma inorganic fluoride concentrations in neurosurgical patients receiving sevoflurane or isoflurane. Anesth Analg. 1997 Sep;85(3):587–92.

54. Golkowski D, Ranft A, Kiel T, Riedl V, Kohl P, Rohrer G, et al. Coherence of BOLD signal and electrical activity in the human brain during deep sevoflurane anesthesia. Brain Behav. 2017 May 17;7(7):e00679.

55. Lam AM, Matta BF, Mayberg TS, Strebel S. Change in cerebral blood flow velocity with onset of EEG silence during inhalation anesthesia in humans: evidence of flow-metabolism coupling? J Cereb Blood Flow Metab Off J Int Soc Cereb Blood Flow Metab. 1995 Jul;15(4):714–7.

56. Reinsfelt B, Westerlind A, Houltz E, Ederberg S, Elam M, Ricksten SE. The effects of isoflurane-induced electroencephalographic burst suppression on cerebral blood flow velocity and cerebral oxygen extraction during cardiopulmonary bypass. Anesth Analg. 2003 Nov;97(5):1246–50.

57. Reinsfelt B, Westerlind A, Ricksten SE. The effects of sevoflurane on cerebral blood flow autoregulation and flow-metabolism coupling during cardiopulmonary bypass. Acta Anaesthesiol Scand. 2011 Jan;55(1):118–23.

58. Akbik F, Robertson M, Das AS, Singhal T, Lee JW, Vaitkevicius H. The PET Sandwich: Using Serial FDG-PET Scans with Interval Burst Suppression to Assess Ictal Components of Disease. Neurocrit Care. 2020 Dec;33(3):657–69.

59. Matta BF, Mayberg TS, Lam AM. Direct cerebrovasodilatory effects of halothane, isoflurane, and desflurane during propofol-induced isoelectric electroencephalogram in humans. Anesthesiology. 1995 Nov;83(5):980–5; discussion 27A.

60. Matta BF, Lam AM. Nitrous oxide increases cerebral blood flow velocity during pharmacologically induced EEG silence in humans. J Neurosurg Anesthesiol. 1995 Apr;7(2):89–93.

61. BF M, KJ H, K T, AC S. Direct cerebral vasodilatory effects of sevoflurane and isoflurane. Vol. 91, Anesthesiology. Department of Anaesthesia, Addenbrooke’s Hospital, Cambridge, United Kingdom. basil@bmatta.demon.co.uk; 1999. p. 677–80.

62. Woodcock TE, Murkin JM, Farrar JK, Tweed WA, Guiraudon GM, McKenzie FN. Pharmacologic EEG suppression during cardiopulmonary bypass: cerebral hemodynamic and metabolic effects of thiopental or isoflurane during hypothermia and normothermia. Anesthesiology. 1987 Aug;67(2):218–24.

63. Zanatta P, Toffolo GM, Sartori E, Bet A, Baldanzi F, Agarwal N, et al. The human brain pacemaker: Synchronized infra-slow neurovascular coupling in patients undergoing non-pulsatile cardiopulmonary bypass. NeuroImage. 2013 May 15;72:10–9.

64. Chalia M, Lee CW, Dempsey LA, Edwards AD, Singh H, Michell AW, et al. Hemodynamic response to burst-suppressed and discontinuous electroencephalography activity in infants with hypoxic ischemic encephalopathy. Neurophotonics. 2016 Jul;3(3):031408.

65. Du B, Shan A, Zhang Y, Zhong X, Chen D, Cai K. Zolpidem arouses patients in vegetative state after brain injury: quantitative evaluation and indications. Am J Med Sci. 2014 Mar;347(3):178–82.

66. Kassab A, Hinnoutondji Toffa D, Robert M, Lesage F, Peng K, Khoa Nguyen D. Hemodynamic changes associated with common EEG patterns in critically ill patients: Pilot results from continuous EEG-fNIRS study. NeuroImage Clin. 2021;32:102880.
